# Angiotensin II type 1a receptor deficiency alleviates muscle atrophy after denervation

**DOI:** 10.1038/s41598-023-27737-7

**Published:** 2023-01-10

**Authors:** Suguru Takayama, Kazuho Inoue, Yuji Ogura, Seiko Hoshino, Takeshi Sugaya, Keiichi Ohata, Hitoshi Kotake, Daisuke Ichikawa, Minoru Watanabe, Kenjiro Kimura, Yugo Shibagaki, Atsuko Kamijo-Ikemori

**Affiliations:** 1 Division of Nephrology and Hypertension, Department of Internal Medicine, St. Marianna University School of Medicine, 2-16-1 Sugao, Miyamae-Ku, Kawasaki, 216-8511 Japan; 2 Department of Anatomy, St. Marianna University School of Medicine, Kanagawa, Japan; 3 Department of Physiology, St. Marianna University School of Medicine, Kanagawa, Japan; 4 Institute for Animal Experimentation, St. Marianna University Graduate School of Medicine, Kanagawa, Japan; 5 JCHO Tokyo Takanawa Hospital, Tokyo, Japan

**Keywords:** Skeletal muscle, Ubiquitin ligases, Apoptosis

## Abstract

The study aim was to determine if suppressed activation of angiotensin II type 1 receptor (AT1) prevents severe muscle atrophy after denervation. The sciatic nerves in right and left inferior limbs were cut in AT1a knockout homo (AT1a^−/−^) male mice and wild-type (AT1a^+/+^) male mice. Muscle weight and cross-sectional areas of type IIb muscle fibers in gastrocnemius muscle decreased at 7 and 21 days postdenervation in both AT1a^−/−^ mice and AT1a^+/+^ mice, and the reduction was significantly attenuated in the denervated muscles of AT1a^−/−^ mice compared to the AT1a^+/+^ mice. Gene expressions in the protein degradation system [two E3 ubiquitin ligases (muscle RING-finger protein-1 and Atrogin-1)] upregulated at 7 days postdenervation in all denervated mice were significantly lower in AT1a^−/−^ mice than in AT1a^+/+^ mice. Activations of nuclear factor κB and Forkhead box subgroup O1, and protein expression of monocyte chemoattractant protein-1 were significantly suppressed in the AT1a^−/−^ mice compared with those in the AT1a^+/+^ mice. In addition, suppressed apoptosis, lower infiltration of M1 macrophages, and higher infiltration of M2 macrophages were significantly observed at 21 days postdenervation in the AT1a^−/−^ mice compared with those in the AT1a^+/+^ mice. In conclusion, the AT1 receptor deficiency retarded muscle atrophy after denervation.

## Introduction

Kidney or heart diseases are known high-risk diseases for sarcopenia defined as a decrease in muscle power and volume^1–3^. Since sarcopenia easily leads to frailty, which is associated with poor prognosis^4–6^, the maintenance of physical function for prevention of sarcopenia should be one goal in clinical management of these diseases. Recently, exercise or increased physical activity have been found to help prevent not only muscle weakness, but also disease progression^7,8^. However, critical comorbidities, such as cardiopulmonary insufficiency or orthopedic disorders, do not allow patients to exercise enough. Therefore, therapeutic strategies for prevention of muscle weakness are needed.

Given that angiotensin II (Ang II) produced by activation of the renin–angiotensin system (RAS) is related to the pathophysiology of kidney or heart diseases via the Ang II type 1 (AT1) receptor, the Ang II signaling blockade has a central role in their management^9,10^. In addition, an unfavorable effect of chronic activated RAS and a beneficial effect of the Ang II signaling blockade on skeletal muscle have been reported in various studies^11–15^. Some experimental studies have shown that AT1 receptor blocker prevented muscle fibrosis and encouraged muscle regeneration after a direct injury of skeletal muscle using freezing^16^, laceration^17^, and cardiotoxin^18^. Furthermore, deletion of the *Agtr1a* gene encoding the AT1a receptor, which is the main mouse isoform of the AT1 receptor and mouse homolog of the single human *AGTR1* gene^19,20^, has been reported to significantly prevent aging-related fibrosis of skeletal muscle^16^. From this evidence, it is possible that inactivation of the AT1 receptor may protect against muscle wasting. However, its usefulness in a disuse model with severe muscle atrophy, which mimics muscle wasting due to kidney or heart diseases that induce chronic and progressive muscle atrophy, has not been sufficiently investigated.

Denervation of the sciatic nerve induces activation of the protein degradation system and results in remarkable loss of muscle mass^21,22^. The present study aim was to determine if inactivation of the AT1a receptor has beneficial effects on severe muscle atrophy after denervation of the sciatic nerve in AT1a receptor knockout homozygous (AT1a^−/−^) male mice. The study results showed that deletion of the AT1a receptor could be associated with attenuation of muscle atrophy due to denervation. Inactivation of the Ang II type 1 receptor may be a useful strategy for retardation of progressive muscle wasting.

## Results

### AT1a receptor loss alleviated denervation-induced muscle atrophy

We used a sciatic nerve denervation model to induce muscle atrophy in 10- to 12-week-old AT1a^−/−^ and AT1a^+/+^ mice. Leg muscle tissue samples were collected at 7 days and 21 days after the denervation procedure (Den group). In sham-operated mice as a control group (Cont group), the leg muscle tissue samples were obtained at 21 days after the sham operation.

#### Animal characteristics

The body weights measured before each denervation and tissue harvest were significantly higher in the AT1a^−/−^-Cont group than in the AT1a^+/+^-Cont group. Body weights were significantly lower in the AT1a^+/+^ mice at 7 days postdenervation and in the AT1a^−/−^ mice at 7 and 21 days postdenervation compared with each control group of the same mice. The body weight levels at 7 days postdenervation were significantly higher in the AT1a^−/−^ mice than those in the AT1a^+/+^ mice (Table 1).

**Table 1:** Animal characteristics at 7 and 21 days postdenervation.

	AT1a^+/+^-Cont (n = 6)	AT1a^+/+^-Den, 7 days (n = 7)	AT1a^+/+^-Den, 21 days (n = 7)	AT1a^−/−^-Cont (n = 5)	AT1a^−/−^-Den, 7 days (n = 6)	AT1a^−/−^-Den, 21 days (n = 6)
Body weight (g) pre-operation	24.6 ± 0.31	24.5 ± 0.42	24.3 ± 0.39	28.0 ± 0.88^##^	26.7 ± 0.63	25.5 ± 0.97
Body weight (g) pre-tissue harvest	27.8 ± 0.47	25.0 ± 0.42**	26.0 ± 0.46	30.1 ± 1.03^#^	27.2 ± 0.65**^, §^	26.5 ± 0.89**
Grip strength (N)	2.49 ± 0.08	2.63 ± 0.07	2.45 ± 0.08	2.75 ± 0.07^#^	2.79 ± 0.03	2.57 ± 0.09
Grip strength (N)/body weight (kg)	104.39 ± 2.58	110.43 ± 3.24	103.94 ± 3.37	99.48 ± 2.19	103.49 ± 1.87	99.16 ± 3.22
Skeletal muscles (mg)						
Gastrocnemius	144.68 ± 2.25	89.67 ± 1.10**	50.82 ± 0.71**	135.14 ± 2.53^#^	93.95 ± 3.86**	53.18 ± 2.77**
Tibialis anterior	52.77 ± 0.57	39.23 ± 0.64**	25.49 ± 0.10**	55.90 ± 1.39	42.12 ± 1.43**	25.16 ± 1.53**
Soleus	8.53 ± 0.43	6.78 ± 0.17**	5.21 ± 0.13**	8.38 ± 0.41	6.78 ± 0.17**	5.63 ± 0.40**
Skeletal muscles (mg)/body weight (g)						
Gastrocnemius	5.22 ± 0.12	3.60 ± 0.10**	1.96 ± 0.03**	4.50 ± 0.07^##^	3.45 ± 0.08**	2.00 ± 0.05**
Tibialis anterior	1.90 ± 0.03	1.57 ± 0.03**	0.98 ± 0.04**	1.87 ± 0.06	1.55 ± 0.03**	0.95 ± 0.03**
Soleus	0.31 ± 0.02	0.25 ± 0.01**	0.20 ± 0.01**	0.28 ± 0.01	0.25 ± 0.001	0.21 ± 0.01**

Values are means ± SE. **P < 0.01 vs the same group control; ^#^*P* < 0.05 and ^##^*P* < 0.01 vs AT1a^+/+^-Cont; ^§^*P* < 0.05 vs AT1a^+/+^-Den (7 days).

Grip strength measured before denervation was significantly higher in the AT1a^−/−^-Cont group than in the AT1a^+/+^-Cont group, but there was no significant difference in grip strength normalized to the body weight (Table 1). In addition, there were no significant differences in grip strength and grip strength normalized to the body weight among the same mice (Table 1).

#### Change in skeletal muscle weight

The skeletal muscle specimens extracted at 7 and 21 days after the procedure were sectioned into the gastrocnemius and tibialis anterior (TA) muscles, as fast muscle, and the soleus muscle, as a slow muscle. In the Cont group, the muscle weight and muscle weight normalized to the body weight in the gastrocnemius muscle were significantly lower in the AT1a^−/−^ mice than in the AT1a^+/+^ mice (Table 1). After denervation, significantly reduced skeletal muscle weight and normalized muscle weight by body weight were evident in the gastrocnemius and TA muscles at 7 and 21 days postdenervation compared with each Cont group in the AT1a^+/+^ and AT1a^−/−^ mice (Table 1). In the soleus muscle of both the AT1a^+/+^ and AT1a^−/−^ mice, the muscle weight at 7 and 21 days postdenervation and the normalized muscle weight at 21 days postdenervation were significantly lower in the Den group than in the Cont group (Table 1). The normalized muscle weight in the soleus muscle at 7 days postdenervation was significantly lower in the Den group than in the Cont group in the AT1a^+/+^ mice, but not in the AT1a^−/−^ mice (Table 1).

**Figure 1:**
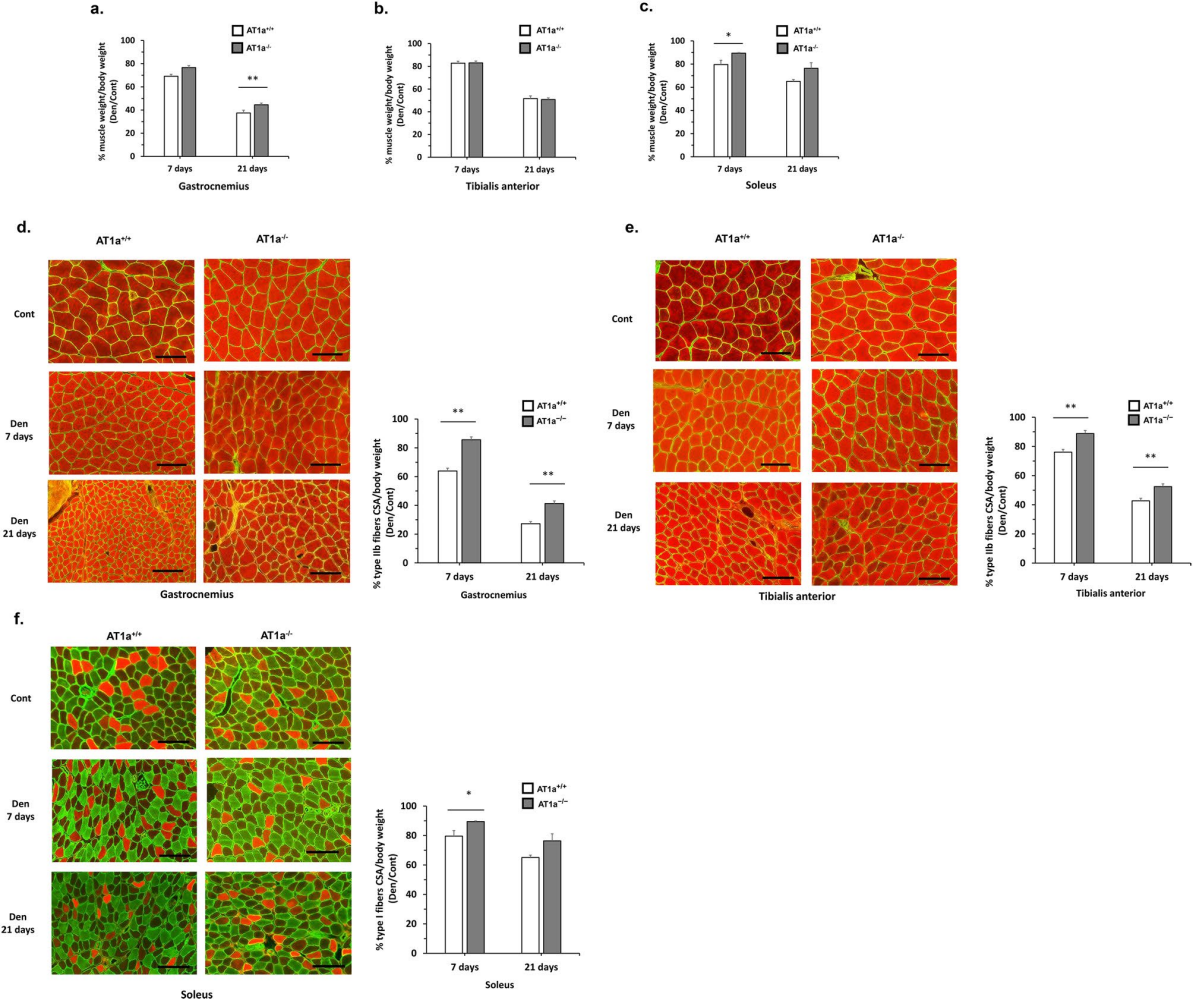
AT1a receptor loss alleviated denervation-induced muscle atrophy. (**a**–**c**) Change in skeletal muscle weight per body weight in denervated gastrocnemius (**a**), tibial anterior (**b**), soleus (**c**) muscles at 7 and 21 days postdenervation. The graphs show the fold decrease in each muscle weight normalized to body weight in denervated muscle (Den group) compared to sham-operated group as control (Cont group). (**d–f**) Immunohistochemistry analysis of muscle fibers. Triple staining with type IIb muscle fibers stained red, type I muscle fibers stained green, and laminin stained green. Cross-section of muscle fibers in gastrocnemius (**d**), tibial anterior (**e**), and soleus (**f**). Scale bar: 100 μm. The graphs show the fold decrease in each cross-sectional area normalized to body weight in denervated muscle (Den group) compared to sham-operated group as control (Cont group). AT1a^+/+^-Cont group, n = 6; AT1a^+/+^-Den group at 7 days postdenervation, n = 7; AT1a^+/+^-Den group at 21 days postdenervation, n = 7; AT1a^−/−^-Cont group, n = 5; AT1a^−/−^-Den group at 7 days postdenervation, n = 6; AT1a^−/−^-Den group at 21 days postdenervation, n = 6. Values are means ± SE. *P < 0.05, **P < 0.01.

The skeletal muscle weights normalized to body weights after denervation are shown as the fold decrease in the Den group compared with the Cont group (Fig. 1a–c). The fold decreases in the normalized gastrocnemius muscle weight in the Den group were observed at 7 days and at 21 days postdenervation (Fig. 1a). The fold decrease in the gastrocnemius muscle at 21 days postdenervation was significantly lower in the AT1a^−/−^ mice compared with AT1a^+/+^ mice (*P* < 0.01, Fig. 1a). In the TA muscle, the fold decreases in the normalized muscle weight in the Den group were observed at 7 days and 21 days postdenervation (Fig. 1b). In the soleus muscle, the fold decreases in the normalized muscle weight in the Den group were observed at 7 days and at 21 days postdenervation (Fig. 1c). The fold decrease in the soleus muscle weight at 7 days postdenervation was significantly lower in the AT1a^−/−^ mice than in the AT1a^+/+^ mice (*P* < 0.05), but not at 21 days postdenervation (Fig. 1c).

#### Cross-sectional area of skeletal muscle fiber

The mouse gastrocnemius and TA muscles are primarily composed of type IIb (fast-twitch) muscle fibers, which are markedly sensitive to denervation-induced atrophy^23^. The mouse soleus muscles are primarily composed of type I (slow-twitch) muscle fibers. These fibers were identified using immunostaining for skeletal muscle myosin; type IIb muscle fibers stained red (Fig. 1d,e), and type I muscle fibers stained green (Fig. 1f). The cross-sectional areas of the type IIb muscle fibers were evaluated in gastrocnemius (Fig. 1d) and TA muscles (Fig. 1e), whereas the type I muscle fibers were evaluated in the soleus muscle (Fig. 1f). The fold decreases in their cross-sectional areas normalized to body weight in the Den group compared with the Cont group are shown in Fig. 1d–f.

In the gastrocnemius muscle, the fold decreases in the normalized cross-sectional areas of type IIb muscle fibers at 7 days (36% and 14% in AT1a^+/+^ and AT1a^−/−^ mice, respectively) and 21 days (73% and 59% in AT1a^+/+^ and AT1a^−/−^ mice, respectively) postdenervation were significantly lower in the AT1a^−/−^ mice than in the AT1a^+/+^ mice (*P* < 0.01, Fig. 1d). In the TA muscle, the fold decreases in the normalized cross-sectional areas of type IIb muscle fibers at 7 days (24% and 11% in AT1a^+/+^ and AT1a^−/−^ mice, respectively) and 21 days (57% and 48% in AT1a^+/+^ and AT1a^−/−^ mice, respectively) postdenervation were significantly lower in the AT1a^−/−^ mice than in the AT1a^+/+^ mice (*P* < 0.01, Fig. 1e). In contrast, in the soleus muscle, the fold decreases in the normalized cross-sectional areas of type I muscle fibers at 7 days postdenervation (20% and 11% in AT1a^+/+^ and AT1a^−/−^ mice, respectively) were significantly lower in the AT1a^−/−^ mice than in the AT1a^+/+^ mice (*P* < 0.05), but not at 21 days postdenervation (35% and 24% in AT1a^+/+^ and AT1a^−/−^ mice, respectively) (Fig. 1f).

These results suggest that AT1a receptor loss was more resistant to fast-twitch (rich in type IIb muscle fiber) muscle atrophy after denervation than to slow-twitch (rich in type I muscle fiber) muscle atrophy. Furthermore, the fold decreases in both normalized muscle weight and cross-sectional areas of type IIb muscle fibers in the gastrocnemius muscle at 21 days postdenervation were significantly attenuated in AT1a^−/−^ mice compared with AT1a^+/+^ mice. Therefore, to elucidate the mechanism underlying the beneficial effects of AT1a receptor loss against the muscle atrophy, the gastrocnemius muscle was used in this study.

### AT1a receptor loss reduced the gene expressions related to protein degradation system through downregulation of FoxO1 expression in the denervated gastrocnemius muscle

#### Gene expressions related to the protein degradation system

Muscle-specific ubiquitin ligases, muscle RING-finger protein-1 (MuRF1) and muscle-specific F-box protein (Atrogin-1) are critical regulators of ubiquitination in atrophying muscles and are related to activation of muscle protein degradation system^24,25^. Therefore, the gene expressions of both *MuRF1* and *Atrogin-1* were evaluated using real-time quantitative reverse transcription polymerase chain reaction (RT-qPCR). The expression levels of these transcripts in each sample were normalized to *18S* ribosomal RNA (rRNA) expression levels and were shown as the fold increase or decrease in mRNA expression in the Den group compared with the Cont group.

**Figure 2:**
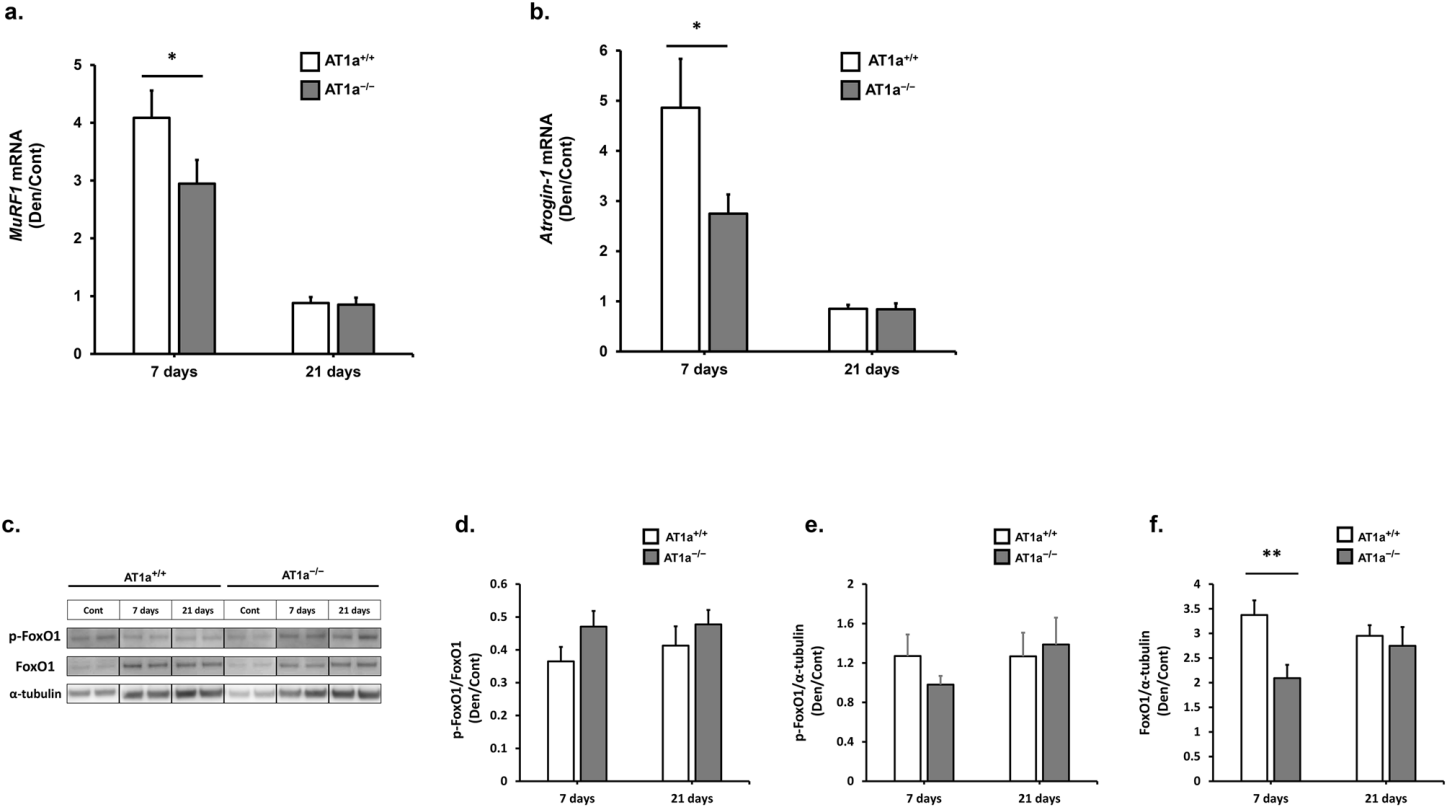
AT1a receptor loss reduced the gene expressions related to protein degradation system through downregulation of FoxO1 expression in the denervated gastrocnemius muscle. (**a**, **b**) Gene expressions of *MuRF1* (**a**) and *Atrogin-1* (**b**) were evaluated by RT-qPCR. Expression levels of these transcripts in each sample were normalized to *18S* rRNA expression levels and were shown as the fold increase or decrease in mRNA expression in the Den group compared with the Cont group. (**c–f**) Protein expression levels of phosphorylated FoxO1 and total FoxO1 were evaluated by western blot analysis. Expression levels in each sample were normalized to α-tubulin expression levels and were shown as the fold increase or decrease in the protein expression in the Den group compared with the Cont group. Relative protein expression was calculated for p-FoxO1/FoxO1 (**d**), p-FoxO1/α-tubulin (**e**), FoxO1/α-tubulin (**f**). Samples from the same experiment were processed in parallel for SDS-PAGE and western blotting using different gels and membranes, and the image data obtained were cropped. Entire images of western blotting are shown in online supplementary resource Supplementary Fig. S5. AT1a^+/+^-Cont group, n = 6; AT1a^+/+^-Den group at 7 days postdenervation, n = 7; AT1a^+/+^-Den group at 21 days postdenervation, n = 7; AT1a^−/−^-Cont group, n = 5; AT1a^−/−^-Den group at 7 days postdenervation, n = 6; AT1a^−/−^-Den group at 21 days postdenervation,n = 6. Values are means ± SE. ^*^P < 0.05, ^**^P < 0.01.

In the AT1a^+/+^ and AT1a^−/−^ mice, the significant upregulated gene expressions of both *MuRF1* (Fig. 2a) and *Atrogin-1* (Fig. 2b) in the denervated gastrocnemius muscle were observed at 7 days postdenervation compared with the Cont group, whereas there were no upregulations of their genes in the denervated gastrocnemius muscle at 21 days postdenervation (Fig. 2a,b). Remarkably, the gene expression levels of both *MuRF1* (Fig. 2a) and *Atrogin-1* (Fig. 2b) that were upregulated at 7 days postdenervation were significantly lower in AT1a^−/−^ mice than in AT1a^+/+^ mice (*P* < 0.05), suggesting that the inactivation of *MuRF1* and *Atrogin-1* due to AT1a receptor loss was responsible for resistance to atrophy after denervation.

#### Inactivation of FoxO1 pathway

The Forkhead box subgroup O (FoxO) family are important regulators of *MuRF1* and *Atrogin-1* gene expression^26^. When phosphorylated, FoxO resides in the cytosol and requires dephosphorylation to enter the nucleus to promote the expressions of both MuRF1 and Atrogin-1^27^. Although the inductions of both MuRF1and Atrogin-1 are normally blocked by phosphorylation of FoxO1 proteins, a subgroup of FoxO family, dephosphorylated FoxO1, may be primarily involved in muscle atrophy^22^.

Therefore, we evaluated the activation of FoxO1 in denervated gastrocnemius muscle by using western blot analysis (Fig. 2c–f). The protein expression levels of phosphorylated FoxO1 (Fig. 2e) and total FoxO1 (Fig. 2f) in each sample were normalized to α-tubulin expression levels and shown as the fold increase or decrease in protein expression in the Den group compared with the Cont group. The ratios of phosphorylated FoxO1 to total FoxO1 were decreased by denervation in the AT1a^+/+^ and AT1a^−/−^ mice and were not significantly different between the AT1a^+/+^ and AT1a^−/−^ mice (Fig. 2d). In contrast, the protein expression levels of FoxO1 were upregulated in the Den group compared with the Cont group in the AT1a^+/+^ and AT1a^−/−^ mice (Fig. 2f) and were significantly lower in AT1a^−/−^ mice than in AT1a^+/+^ mice at 7 days postdenervation (*P* < 0.01). Upregulation of FoxO1 protein expression was also observed at 21 days postdenervation in the AT1a^+/+^ and AT1a^−/−^ mice, but there were no significant differences between the AT1a^−/−^ and AT1a^+/+^ mice (Fig. 2f).

### AT1a receptor loss mitigated apoptosis in the denervated gastrocnemius muscle

**Figure 3:**
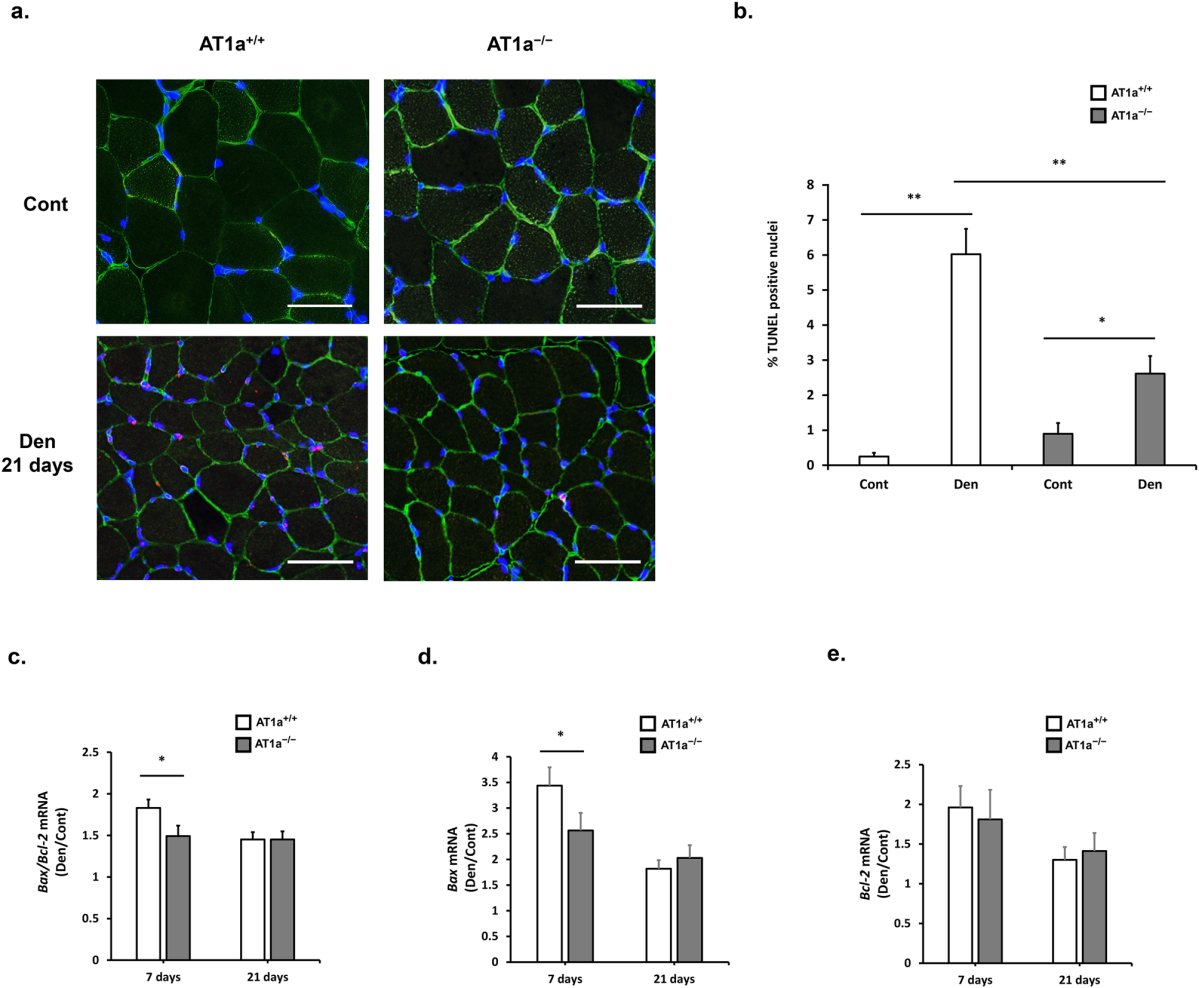
AT1a receptor loss mitigated apoptosis in the denervated gastrocnemius muscle. (**a**) Immunostaining of gastrocnemius muscles in each AT1a^+/+^ and AT1a^−/−^ mice at 21 days postdenervation. Apoptotic cells were detected by TdT-mediated dUTP nick-end-labeling (TUNEL) staining in the denervated gastrocnemius muscles. Triple staining with TUNEL positive apoptotic nuclei (red), nuclear stained by DAPI (blue), and laminin (green). Scale bar: 50 μm. (**b**) The graphs show the ratio (%) of TUNEL-positive nuclei to all the nuclei (TUNEL- or DAPI-positive nuclei). (**c–e**) Gene expression of *Bax/Bcl-2* ratio (**c**), *Bax* (**d**), and *Bcl-2* (**e**) was evaluated by RT-qPCR. Expression levels of these transcripts in each sample were normalized to *18S* rRNA expression levels and were shown as the fold increase or decrease in mRNA expression in the Den group compared with the Cont group. AT1a^+/+^-Cont group, n = 6; AT1a^+/+^-Den group, n = 7; AT1a^−/−^-Cont group, n = 5; AT1a^−/−^-Den group, n = 6. Values are means ± SE. *P < 0.05, **P < 0.01.

Previously, activation of mitochondria-associated apoptosis has been shown in denervated muscle, which induces a considerable loss of muscle mass^28^. Furthermore, activation of FoxO1 has been reported to promote skeletal muscle atrophy through induction of apoptosis in addition to muscle proteolysis^29^. Therefore, we investigated TdT-mediated dUTP nick-end-labeling (TUNEL) staining to evaluate DNA fragmentation in apoptotic nuclei at 21 days postdenervation in gastrocnemius muscle (Fig. 3a). In both AT1a^+/+^ (*P* < 0.01) and AT1a^−/−^ mice (*P* < 0.05), TUNEL-positive nuclei stained red were mildly but significantly observed in the Den group but not in the Cont group (Fig. 3a,b). The positive rate in the Den group was significantly lower in AT1a^−/−^ mice than in AT1a^+/+^ mice (*P* < 0.01, Fig. 3b).

Next, the gene expressions of pro-apoptotic Bcl-2-associated X protein (Bax) and anti-apoptotic B-cell/CLL lymphoma 2 (Bcl-2) were evaluated by using RT-qPCR. Expression levels of these transcripts in each sample were normalized to *18S* rRNA expression levels and were shown as the fold increase or decrease in mRNA expression in the Den group compared with the Cont group (Fig. 3c–e). Although denervation significantly upregulated the gene expressions of *Bax* at 7 and 21 days postdenervation (Fig. 3d) and *Bcl-2* at 7 days postdenervation (Fig. 3e) in both AT1a^+/+^ and AT1a^−/−^ mice, the gene expression levels of *Bax* (*P* < 0.05, Fig. 3d) and the *Bax/Bcl-2* ratio (*P* < 0.05, Fig. 3c) were significantly reduced in the AT1a^−/−^ mice compared with the AT1a^+/+^ mice at 7 days postdenervation. These results suggest that AT1a receptor loss mitigates apoptosis induced by denervation.

### AT1a receptor loss modulated M1/M2 macrophage polarization in the denervated gastrocnemius muscle

**Figure 4:**
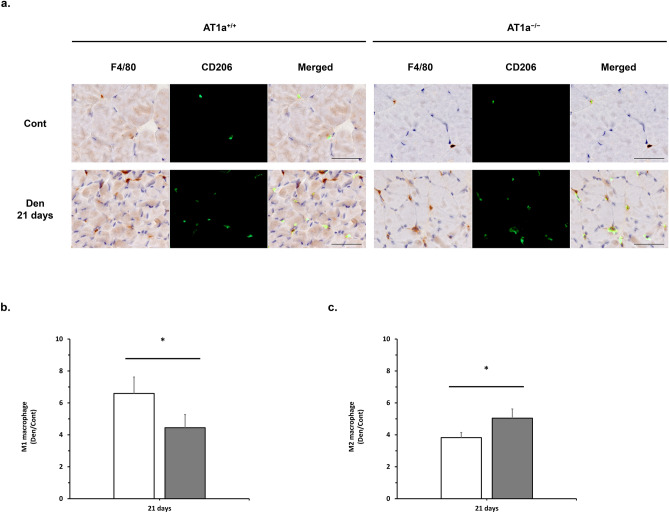
AT1a receptor loss modulated M1/M2 macrophage polarization in the denervated gastrocnemius muscle. (**a**) Immunostaining of gastrocnemius muscle in each AT1a^+/+^ and AT1a^−/−^ mice at 21 days postdenervation. Immunostaining shows codistribution of M1 and M2 macrophages. Anti-F4/80 antibody binds M1 macrophages and M2 macrophages (brown color). Anti-CD206 antibody binds M2 macrophages (green fluorophore). CD206^-^/F4/80^+^ cells are M1 macrophages. CD206^+^/F4/80^+^ cells are M2 macrophages. Scale bar: 50 μm. (**b**, **c**) The degrees of infiltrated M1 macrophage (**b**) and M2 macrophage (**c**) were shown as the fold increase or decrease in the Den group compared with the Cont group in immunohistochemistry analysis. AT1a^+/+^-Cont group, n = 6; AT1a^+/+^-Den group, n = 7; AT1a^−/−^-Cont group, n = 5; AT1a^−/−^-Den group, n = 6. Values are means ± SE. ^*^P < 0.05.

Macrophages have been classified into two polarization states, proinflammatory M1 macrophage and anti-inflammatory M2 macrophage^30^. It has previously been reported that infiltration of M1 macrophage exacerbates muscle atrophy after peripheral nerve injury^31^. AT1a receptor of bone marrow-derived macrophages also worsens the extent of atherosclerosis by shifting the macrophage phenotype to more M1 and less M2 through mechanisms that include increased apoptosis^32^. Therefore, we investigated M1/M2 macrophage polarization in the denervated gastrocnemius muscle in immunohistochemical analysis using anti-F4/80 antibody and anti-CD206 antibody (Fig. 4a). CD206^-^/F4/80^+^ cells as M1 macrophages and CD206^+^/F4/80^+^ cells as M2 macrophages were evaluated quantitatively. The degrees of infiltrated M1 macrophage (Fig. 4b) and M2 macrophage (Fig. 4c) were shown as the fold increase or decrease in the Den group compared with the Cont group.

In the AT1a^+/+^ and AT1a^−/−^ mice, both M1 and M2 macrophage accumulation were elevated in the denervated gastrocnemius muscle at 21 days postdenervation compared with the Cont group (Fig. 4a–c). Remarkably, the elevation of M1 macrophage accumulation were significantly lower in AT1a^−/−^ mice than in AT1a^+/+^ mice (*P* < 0.05, Fig. 4b), on the other hands, the elevation of M2 macrophage accumulation were significantly higher in AT1a^−/−^ mice than in AT1a^+/+^ mice (*P* < 0.05, Fig. 4c). These results suggested that AT1a receptor loss was related to modulation of M1/M2 polarization.

### AT1a receptor loss alleviated denervation-induced muscle atrophy independently of C1q/Wnt/β-catenin signaling in gastrocnemius muscle

**Figure 5:**
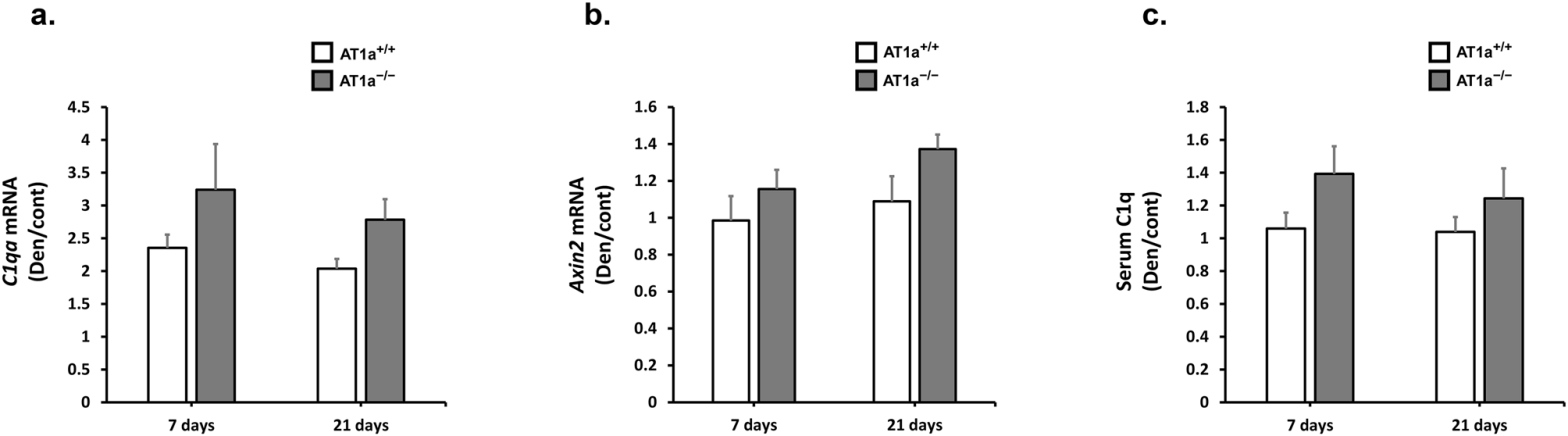
AT1a receptor loss alleviated denervation-induced muscle atrophy independently of C1q/Wnt/β-catenin signaling in gastrocnemius muscle. (**a**, **b**) Gene expressions of *C1qa* (**a**) and *Axin2* (**b**) were evaluated by RT-qPCR. Expression levels of these transcripts in each sample were normalized to *18S* rRNA expression levels and were shown as the fold increase or decrease in mRNA expression in the Den group compared with the Cont group. (**c**) Serum C1q concentration was measured by ELISA. The graphs show the fold increase or decrease in serum concentration levels in the Den group compared with the Cont group. AT1a^+/+^-Cont group, n = 6; AT1a^+/+^-Den group at 7 days postdenervation, n = 7; AT1a^+/+^-Den group at 21 days postdenervation, n = 7; AT1a^−/−^-Cont group, n = 5; AT1a^−/−^-Den group at 7 days postdenervation, n = 6; AT1a^−/−^-Den group at 21 days postdenervation, n = 6. Values are means ± SE.

Previously, AT1 receptor blockade was reported to provide the positive effects against muscle injury via inactivation of complement C1q/Wnt/β-catenin signaling^16^. Therefore, we evaluated the gene expression levels of *C1qa* and *Axin2*, which is a downstream molecule in the Wnt/β-catenin signaling pathway, in denervated gastrocnemius muscle, and measured serum complement C1q. The expression levels of these transcripts in each sample were normalized to *18S* rRNA expression levels and shown as the fold increase or decrease in mRNA expression in the Den group compared with the Cont group (Fig. 5a,b). Serum C1q was shown as the fold increase or decrease in Serum C1q concentration in the Den group compared with the Cont group (Fig. 5c).

Although denervation significantly upregulated the gene expressions of complement *C1qa* at 7 and 21 days postdenervation in both AT1a^+/+^ and AT1a^−/−^ mice, the expressions were not significantly different between the AT1a^+/+^ and AT1a^−/−^ mice (Fig. 5a). In addition, the gene expressions of *Axin2* at 7 and 21 days postdenervation were not significantly upregulated by denervation in both AT1a^+/+^ and AT1a^−/−^ mice (Fig. 5b). Serum C1q concentration was not significantly increased by denervation in both AT1a^+/+^ and AT1a^−/−^ mice (Fig. 5c).

### Denervation did not upregulate the gene expressions of *AT1a receptor* (*Agtr1a*) and *angiotensinogen* in gastrocnemius muscle of AT1a^+/+^ mice

**Figure 6:**
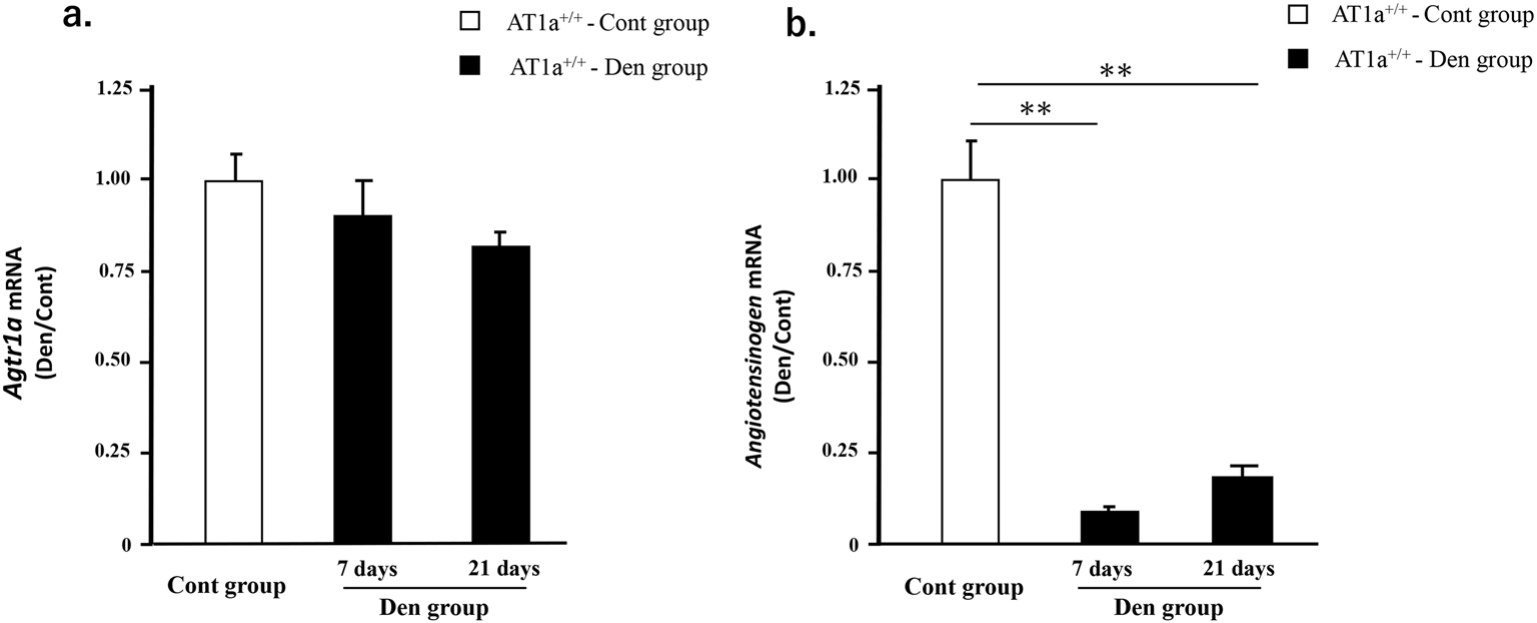
Denervation did not upregulate the gene expressions of *AT1a receptor* (*Agtr1a*) and *angiotensinogen* in gastrocnemius muscle of AT1a^+/+^ mice. (**a**, **b**) Gene expressions of *Agtr1a* (**a**) and *angiotensinogen* (**b**) in gastrocnemius muscle were evaluated by RT-qPCR in AT1a^+/+^ mice. Expression levels of these transcripts in each sample were normalized to *18S* rRNA expression levels and were shown as the fold increase or decrease in mRNA expression in the Den group compared with the Cont group in mice. AT1a^+/+^-Cont group, n = 6; AT1a^+/+^-Den group at 7 days postdenervation, n = 7; AT1a^+/+^-Den group at 21 days postdenervation, n = 7. Values are means ± SE. **P < 0.01.

The renin-angiotensin system (RAS) is a candidate mediator that may promote muscle atrophy^13^. Therefore, we evaluated the gene expressions of *AT1a receptor* (*Agtr1a*) and *angiotensinogen* in gastrocnemius muscle of AT1a^+/+^ mice. The expression levels of these transcripts in each sample were normalized to *18S* rRNA expression levels and shown as the fold increase or decrease in mRNA expression in the Den group compared with the Cont group (Fig. 6a,b). The gene expression of *Agtr1a* was not upregulated and the gene expressions of *angiotensinogen* was rather attenuated in the denervated gastrocnemius muscle.

### AT1a receptor loss reduces proinflammatory response at 3 days postdenervation in denervated gastrocnemius muscle

Because the proinflammatory response has been shown to be related to muscle atrophy^21^, the effects of AT1a receptor loss on the proinflammation in denervated gastrocnemius muscle was evaluated at 3 days postdenervation when muscle atrophy was not brought.

#### Animal characteristics and change in skeletal muscle weight

The levels of body weight measured before denervation were significantly higher in each AT1a^−/−^-Cont and AT1a^−/−^-Den group than in each AT1a^+/+^-Cont and AT1a^+/+^-Den group (*P* < 0.01, Table 2). At 3 days postdenervation, the body weights were significantly higher in the AT1a^−/−^-Den group than in the AT1a^+/+^-Den group (*P* < 0.05, Table 2). The body weights preoperation and at 3 days postdenervation were not significantly different both in the AT1a^+/+^ and AT1a^−/−^ mice compared with each control group of the same mice (Table 2).

**Table 2:** Animal characteristics at 3 days postdenervation.

	AT1a^+/+^-Cont (n = 9)	AT1a^+/+^-Den, 3 days (n = 9)	AT1a^−/−^-Cont (n = 9)	AT1a^−/−^-Den, 3 days (n = 10)
Body weight (g) pre-operation	26.8 ± 0.63	26.4 ± 0.52	29.6 ± 1.06**	30.3 ± 0.58^##^
Body weight (g) pre-tissue harvest	24.7 ± 0.55	24.9 ± 0.58	26.5 ± 0.92	27.3 ± 0.52^#^
Grip strength (N)	2.97 ± 0.1	2.87 ± 0.1	2.83 ± 0.11	2.89 ± 0.09
Grip strength (N)/body weight (kg)	111.31 ± 3.96	108.6 ± 3.07	95.97 ± 3.35*	95.44 ± 2.83^#^
Skeletal muscles (mg)				
Gastrocnemius	136.26 ± 3.00	133.38 ± 2.49	128.51 ± 6.19	133.80 ± 1.92
Tibialis anterior	49.58 ± 1.14	50.98 ± 1.00	49.97 ± 1.97	52.18 ± 0.57
Soleus	7.96 ± 0.19	8.11 ± 0.22	7.68 ± 0.54	7.98 ± 0.16
Skeletal muscles (mg)/body weight (g)				
Gastrocnemius	5.52 ± 0.074	5.36 ± 0.068	4.83 ± 0.086**	4.91 ± 0.093^##^
Tibialis anterior	2.01 ± 0.022	2.05 ± 0.029	1.88 ± 0.022**	1.92 ± 0.034^##^
Soleus	0.32 ± 0.0069	0.33 ± 0.0067	0.29 ± 0.012**	0.29 ± 0.0046^##^

Values are means ± SE. *P < 0.05 and **P < 0.01 vs AT1a^+/+^-Cont; ^#^*P* < 0.05 and ^##^*P* < 0.01 vs AT1a^+/+^-Den (3 days).

Grip strength measured before denervation was not significantly different in all the mice (Table 2), but the grip strength normalized to the body weight was significantly lower in each AT1a^−/−^-Cont and AT1a^−/−^-Den group than in each AT1a^+/+^-Cont and AT1a^+/+^-Den group (*P* < 0.05, Table 2).

The skeletal muscle specimens at 3 days after the procedure were sectioned into the gastrocnemius, TA muscle, and soleus muscle, and their muscle weights were measured. Although none of the sectioned muscle weights were significantly different in the mice, the muscle weights normalized to the body weights in the gastrocnemius, TA, and soleus muscles were significantly lower in each AT1a^−/−^-Cont and AT1a^−/−^-Den groups than in the AT1a^+/+^-Cont and AT1a^+/+^-Den groups (*P* < 0.01, Table 2).

**Figure 7:**
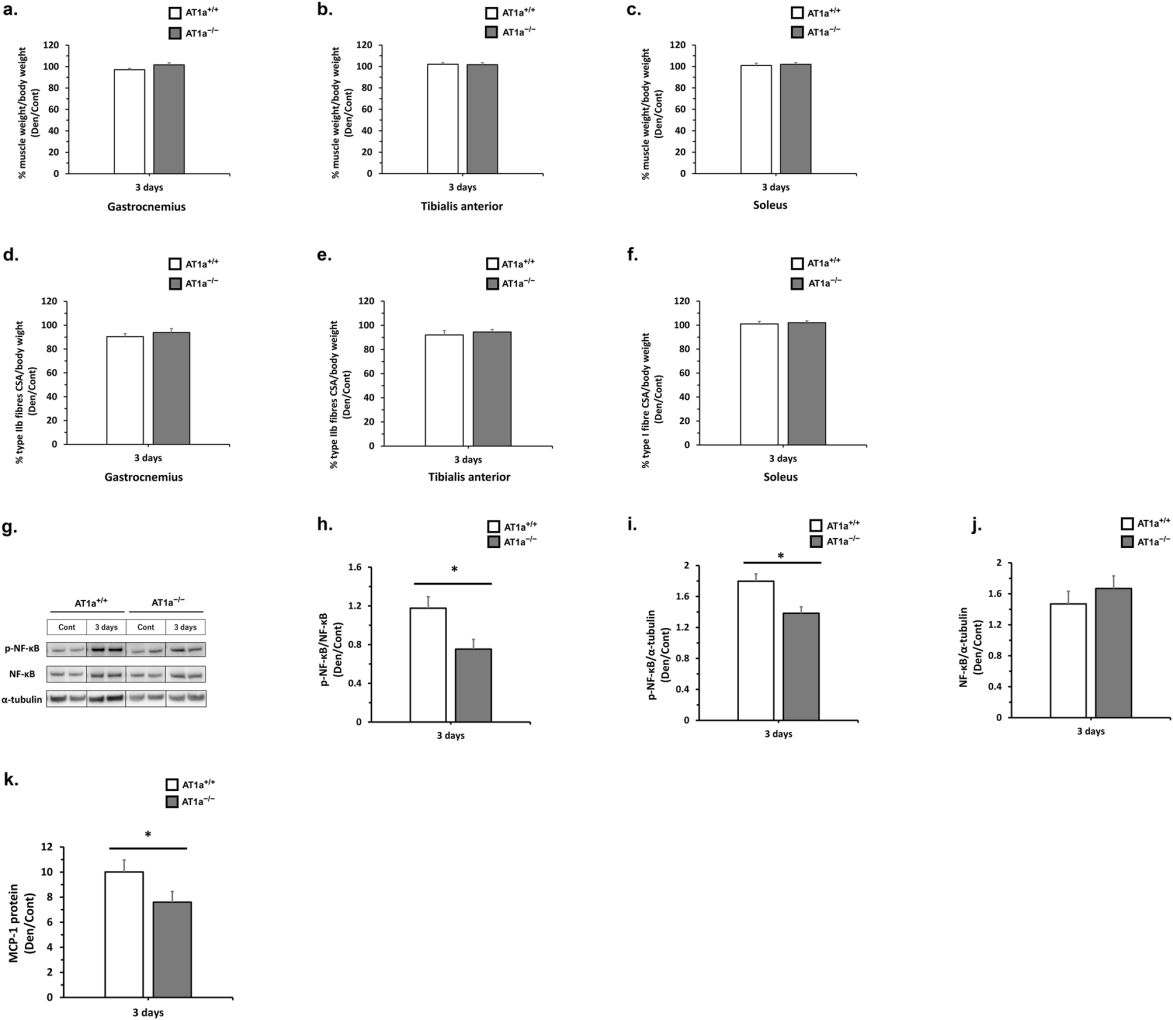
Evaluation of muscle wasting at 3 days postdenervation. (**a–c**) Change in skeletal muscle weight normalized to the body weight in denervated gastrocnemius (**a**), tibial anterior (**b**), and soleus (**c**) muscles at 3 days postdenervation. The graphs show the fold increase or decrease in each normalized muscle weight in the Den group compared with the Cont group. (**d–f**) Quantitative analysis of cross-sectional areas of type IIb and type I muscle fibers normalized to the body weight in gastrocnemius (**d**), tibial anterior (**e**), and soleus muscle (**f**) in the Den group compared with the Cont group at 3 days postdenervation. The graphs show the fold decrease in each cross-sectional area normalized to body weight in the Den group compared to the Cont group. (**g–j**) Protein expression levels of phosphorylated NF-κB and total NF-κB were evaluated by western blot analysis. Expression levels in each sample were normalized to α-tubulin expression levels and were shown as the fold increase or decrease in protein expression in the Den group compared with the Cont group. Relative protein expression was calculated for p-NF-κB/NF-κB (**h**), p-NF-κB/α-tubulin (**i**), and NF-κB/α-tubulin (**j**). Samples from the same experiment were processed in parallel for SDS-PAGE and western blotting using different gels and membranes, and the image data obtained were cropped. Entire images of western blotting are shown in online supplementary resource Supplementary Fig. S6. (**k**) Protein expression levels of MCP-1 were measured by ELISA. The graphs show the fold increase or decrease in protein concentration levels in the Den group compared with the Cont group. The protein concentrations of MCP-1 in each sample were normalized to total protein levels. The graph shows the fold increase in normalized MCP-1 protein in the Den group compared with the Cont group. AT1a^+/+^-Cont group, n = 9; AT1a^+/+^-Den group, n = 9; AT1a^−/−^-Cont group, n = 9; AT1a^−/−^-Den group, n = 10. Values are means ± SE. *P < 0.05.

The skeletal muscle weights normalized to body weights after denervation at 3 days postdenervation are shown as the fold decrease in the Den group compared with the Cont group (Fig. 7a–c). There were no significant fold decreases in any of the normalized muscle weights in the Den group compared with those in the Cont group both in AT1a^+/+^ and AT1a^−/−^ mice, and there were no significant differences between the AT1a^+/+^ and AT1a^−/−^ mice (Fig. 7a–c).

The muscle cross-sectional areas of type IIb and type I muscle fibers at 3 days postdenervation are shown as the fold decrease in their cross-sectional areas normalized to body weight in the Den group compared with the Cont group (Fig. 7d–f). No significant fold decreases in the normalized cross-sectional areas of type IIb and type I muscle fibers were observed in the Den group compared with the Cont group both in AT1a^+/+^ and AT1a^−/−^ mice, and there were no significant differences between the AT1a^+/+^ and AT1a^−/−^ mice (Fig. 7d–f).

#### Evaluation of proinflammatory response, nuclear factor κB, and monocyte chemoattractant protein-1

Nuclear factor κB (NF-κB) transcription factors, which have major roles as mediators of inflammation, have been reported to increase in response to denervation and derive the gene expression of *MuRF1*^21^. Therefore, we evaluated the activation of NF-κB in denervated gastrocnemius muscle by performing western blot analysis (Fig. 7g–j). Protein expression levels of phosphorylated NF-κB and NF-κB in each sample were normalized to α-tubulin expression levels and shown as the fold increase or decrease in protein expression in the Den group compared with the Cont group (Fig. 7i,j). The protein expression levels of phosphorylated NF-κB were upregulated in the Den group compared with the Cont group in both the AT1a^+/+^ and AT1a^−/−^ mice and were significantly lower in AT1a^−/−^ mice than in AT1a^+/+^ mice at 3 days postdenervation (Fig. 7i). The protein expression levels of NF-κB were increased by denervation in both the AT1a^+/+^ and AT1a^−/−^ mice and were not significantly different between the AT1a^+/+^ and AT1a^−/−^ mice (Fig. 7j). The ratios of phosphorylated NF-κB to total NF-κB were significantly lower in the AT1a^−/−^ mice than in the AT1a^+/+^ mice (Fig. 7h).

To evaluate the effects of inflammatory response in denervated muscle, we next evaluated the protein expression of monocyte chemoattractant protein-1 (MCP-1), which is regulated by activation of NF-κB, by enzyme-linked immunosorbent assay (ELISA) (Fig. 7k). The protein concentrations of MCP-1 in each sample were normalized to the total protein levels and shown as the fold increase in normalized MCP-1 protein in the Den group compared with the Cont group (Fig. 7k). The MCP-1 expression levels were significantly upregulated by denervation in both the AT1a^+/+^ and AT1a^−/−^ mice, and notably, these upregulations were significantly lower in the AT1a^−/−^ mice than in the AT1a^+/+^ mice (Fig. 7k).

These results suggested that AT1a receptor loss was resistant to the proinflammatory response caused by the denervated muscle without muscle atrophy.

## Discussion

This study showed that marked reductions in both muscle weight and cross-sectional area of type IIb muscle fibers in gastrocnemius muscle at 21 days postdenervation were induced in AT1a^+/+^ mice, and their reductions were significantly moderated in AT1a^−/−^ mice, whereas there were no significant differences in the degrees of both decreased muscle weights and cross-sectional areas of the type I muscle fibers in soleus muscle at 21 days postdenervation between the AT1a^+/+^ and AT1a^−/−^ mice. The gene expressions of *MuRF1* and *Atrogin-1*, which are related to activation of the protein degradation system, were upregulated at 7 days postdenervation in all of the denervated mice and were significantly lower in AT1a^−/−^ mice than in the AT1a^+/+^ mice. Because phosphorylated NF-κB at 3 days postdenervation and upregulation of FoxO1 expression at 7 days postdenervation were significantly diminished in the gastrocnemius muscle of AT1a^−/−^ mice, AT1a receptor loss may lead to inactivation of the protein degradation system via downregulated phosphorylated NF-κB and FoxO1 expressions. In addition, suppressed apoptosis, lower infiltration of M1 macrophages, and higher infiltration of M2 macrophages at 21 days postdenervation, and suppressed protein expression of MCP-1 at 3 days postdenervation were observed in the AT1a^−/−^ mice compared with those in the AT1a^+/+^ mice. These results indicate that inactivation of the Ang II type 1 receptor might be useful for prevention of muscle atrophy via inactivation of the protein degradation system, apoptosis, and anti-inflammatory action in the disuse model.

Deficiency in the AT1a receptor was reported to be associated with hyperphagia and obesity with increased adiposity^33^, which was supported by our additional experimental study (Supplementary Tables S1 and S2). In contrast, the mean values of grip strength corrected for body weight were lower in the AT1a^−/−^ mice than in the AT1a^+/+^mice. Furthermore, the mean values of muscle weights corrected by body weight in the control group of AT1a^−/−^ mice were lower than those in the control group of the AT1a^+/+^ mice, supporting a previous study finding that deficiency in the AT1a receptor reduced skeletal muscle weight^34^. In the AT1a^−/−^ mice, the body weight before denervation was lower in the Den group than in the control group, and the Den group did not benefit from the body weight more than the control group. Therefore, the beneficial effects of AT1a deficiency against muscle atrophy shown in this study may not have been due to the difference in the body weights between the AT1a^+/+^ and AT1a^−/−^ mice.

Although AT1a receptor loss has been shown to elongate longevity and prevent reduction of muscle strength with aging^16,19^, AT1a receptor loss has been reported to be associated with smaller skeletal muscles^34^, which is consistent with our results. Although the mechanism by which a defect in the AT1a receptor induced smaller skeletal muscle was not clearly demonstrated, our study indicated that the defective AT1a receptor alleviated skeletal muscle atrophy due to denervation. Furthermore, we reconfirmed the positive effects of pharmacological blockade of AT1 receptor, losartan, against the denervated muscle atrophy (Supplementary Fig. S1). In denervation-induced muscle atrophy, activations of NF-κB and FoxO1 have a central role in muscle atrophy via upregulation of the expressions of MuRF1 and Atrogin-1^21,22^. Our results showed that the defective AT1a receptor attenuated the activation of NF-κB and the expression of FoxO1; therefore, the defect was considered to mitigate denervation-induced muscle atrophy via inactivation of both the NF-κB and FoxO1 signaling pathways.

Previous studies have shown that apoptosis via upregulation of pro-apoptotic Bax was induced in denervated muscle^35^ and that Ang II receptor blockade protected against apoptosis and atrophy in skeletal muscle of an experimental heart failure model^36^, which supports our results that deficiency in the AT1a receptor downregulated *Bax* gene expression and decreased the *Bax/Bcl-2* ratio at 7 days postdenervation, and led to a lower number of TUNEL-positive nuclei at 21 days postdenervation. Muscle apoptosis has been reported to be regulated by activation of FoxO1, and the present results showed that the defective AT1a receptor reduced upregulation of FoxO1, as described above. This deficiency in the AT1 receptor may contribute to an anti-apoptotic effect via suppressed FoxO1 expression in the denervation model. Reduction of muscle apoptosis has been reported to prevent denervated muscle atrophy^37^, and the anti-apoptotic effect caused by the AT1a receptor deficiency may be related to attenuation of the denervated muscle atrophy.

A previous study showed that induction of FoxO1 expression was due to the complement C1q/Wnt/β-catenin signaling pathway in muscle wasting with chronic heart failure^38^. Complement C1q, which is secreted by macrophages, was reported to increase in the serum of mice with chronic heart failure^38^ and elderly mice^39,40^ and subsequently activated FoxO signaling by activating Wnt/β-catenin signaling in skeletal muscle. The AT1 receptor blockade has been shown to prevent the production of complement C1q in macrophages that infiltrated muscle and to inactivate the muscular Wnt/β-catenin signaling, resulting in early muscle repair after cryoinjured muscle^16^. In contrast, serum complement C1q levels did not increase in the denervated mice. Although the expression of complement *C1qa* in the denervated muscle was upregulated in the mice with and without AT1a receptor, the degree of expression was similar between the two groups. The gene expression of *Axin2*, which is a downstream gene of Wnt/β-catenin signaling, was not changed by the denervation in the mice with and without AT1a receptor. Although muscle atrophy was rapidly and remarkably generated by the denervation, accumulation of skeletal muscle loss and fibrosis, which were observed in aging and cryoinjured muscle, was not stimulated at 21 days postdenervation. The activation of complement C1q/Wnt/β-catenin signaling may be involved in progression of muscle fibrosis^40^. Furthermore, production of complement C1q occurs at a degree to which increased serum complement C1q levels may be needed for activation of complement C1q/Wnt/β-catenin signaling. In the denervated muscle, the induction of FoxO1 expression was not due to activation of the complement C1q/Wnt/β-catenin signaling pathway, and the beneficial effects of depleted AT1a receptor against the denervated atrophy was independent of the C1q/Wnt/β-catenin signaling pathway.

Regarding the influence of inactivated AT1 receptor on muscle protein synthesis, the AT1 receptor blocker was reported to induce activation of Akt-mTOR signaling, increased protein synthesis, and thereby protect against disuse-related muscle atrophy in aged mice subjected to hindlimb immobilization^18^. Contrary to this result, our results showed that that AT1a receptor loss did not accelerate protein synthesis (Supplementary Fig. S2). These results indicated that the positive effects of the AT1a receptor loss against the denervated muscle atrophy were due to attenuated muscle protein degradation.

Infiltration of M1 macrophages induced by cytokines such as MCP-1 was reported to play a central role for muscle atrophy in the peripheral nerve injury established by the ligation of sciatic nerve^31^. It was reported that the activity of AT1 receptor in macrophages promoted M1 macrophages polarization which accelerated the inflammation with progression of tissue damage and that its suppression leaded to alleviate the inflammation via inducing M2 macrophages polarization^32,41^. The experimental study of muscle cryoinjury using mice reported that the blocking of AT1 receptor might contribute to accelerate muscle repair via the shift in macrophages from M1 to M2 phenotype^16^. Our present study also showed decreased infiltration of M1 macrophages and increased infiltration of M2 macrophages at 21 days postdenervation, and suppressed protein expression of MCP-1 at 3 days postdenervation in the deficiency of AT1a receptor. Therefore, modulation of M1/M2 polarization with anti-inflammatory effects by AT1a receptor loss might contribute to the relief of the denervated muscle atrophy.

AT1a receptor is expressed in satellite cells, the deficiency of AT1a receptor may influence on muscle repair function regulated by activation of muscle satellite cells. Therefore, the gene expressions of *Pax7* which is expressed in activate and proliferative muscle satellite cells, and both *MyoD* and *myogenin* which play critical roles for the differentiation of myoblasts to myofibers in muscle tissue, were evaluated by RT-qPCR. As a result, AT1a receptor deficiency did not show a novel regenerative response against the denervated muscle atrophy (Supplementary Fig. S3). However, there is conflicting evidence on the relationship between AT1aR and muscle regeneration^42^, and further investigations are needed.

Histologically, we showed the protective effects of AT1a receptor deficiency on type IIb muscle fibers of the gastrocnemius and TA muscles, but not on type I muscle fibers of the soleus muscle in denervated muscle. Another study also showed different effects against the denervated muscle atrophy in different muscle fibers^43^. In addition, the muscle atrophy in the soleus muscle was not attenuated by Ang II blockade treatment in an unloading model using male rats^44^. We confirmed the expression of AT1a receptor in the soleus muscle (data not shown) but did not elucidate the mechanism underlying the muscle fiber type-specific effect of AT1a receptor deficiency. Although type I muscle fibers may be treatment resistant, further research is needed to elucidate the mechanism to establish a strategy against sarcopenia in the future.

The results of this study may have been limited by some factors. First, although decreased muscle weights in gastrocnemius and solus evaluated by change ratios of Den group for Cont group were significantly suppressed in AT1a^−/−^ mice compared to AT1a^+/+^ mice (Fig. 1a,b), there were not significant differences in actual measured skeletal muscle weights and those normalized to body weights after denervation between AT1a^−/−^ mice and AT1a^+/+^ mice (Table 1) because there were significant differences in basal body weight and skeletal muscle weight between AT1a^−/−^ mice and AT1a^+/+^ mice. Second, the muscle weight of the mice without AT1a receptor was smaller than that of the mice with the AT1a receptor. AT1a receptor signaling may be important to not only neonatal muscle development, but also to postnatal myogenesis and may mediate the muscle fiber growth. However, our study showed the positive effects of AT1a receptor deficiency against muscle wasting in a mature muscle. AT1a receptor signaling may have different roles in each muscle growth and muscle wasting. Third, muscle strength which reflects muscle function was not able to be evaluated for denervation model in the present study. Fourth, our additional experiment showed that weights of adipose tissue and kidney at 21 days postdenervation were significantly higher in all AT1a^−/−^ mice than in all AT1a^+/+^ mice and the weight of adipose tissue in the AT1a^−/−^ mice was significantly decreased by denervation, but not the AT1a^+/+^ mice (Supplementary Table S2). The degradation of adipose tissue may be induced for conservation of energy source after denervation in the AT1a^−/−^ mice. Further study is needed to reveal whether the difference of lipid utility between AT1a^−/−^ mice and AT1a^+/+^ mice may influence the progression of muscle atrophy. Finally, the activation of RAS in myofibers was reported to be related to muscle atrophy^13^. However, the present study did not show the upregulation of both AT1a receptor and angiotensinogen expressions by denervation in gastrocnemius muscle of the AT1a^+/+^ mice. In addition, the degrees of AT1a receptor expression were lower in skeletal muscles than in other organs except for a brain (Supplementary Fig. S4) and there is a possibility that contribution of AT1a receptor in myofibers to muscle atrophy after denervation may be low. On the other hand, AT1a receptor expresses in various cells such as infiltrated macrophages, sympathetic nerve terminals, and satellite cells, in muscle tissue. The mice without systemic AT1a receptor were used in the present study and thus the cell type-specific AT1a receptor loss is needed to reveal the more detail relationship between AT1a receptor and muscle atrophy in the future study.

In conclusion, the study findings revealed that AT1a receptor loss retarded muscle atrophy after denervation by attenuating the protein degradation system, inactivating both the NF-κB and FoxO1 signaling pathways, and via antiapoptotic action and anti-inflammatory action. Denervation as a disuse model involves common cellular signaling in muscle atrophy related to systemic wasting conditions, such as those associated with kidney or heart diseases^45^. In a clinical study, blockade of Ang II signaling by Ang II type I receptor blocker has been reported to prevent muscle wasting in end-stage kidney disease or congestive heart failure^12,13^. Our preclinical results highlight the usefulness of inactivation of the Ang II type 1 receptor for prevention of muscle wasting.

## Methods

### Animals

This study was conducted according to the St. Marianna University School of Medicine Institutional Guide for Animal Experiments and the Guide for the Care and Use of Laboratory Animals (National Institutes of Health (NIH), Bethesda, MD, USA). This study was reported in accordance with ARRIVE guidelines (https://arriveguidelines.org). All of the experimental protocols in this study were approved by the Ethics Committee on Animal Experiments of the St. Marianna University School of Medicine (Authorization Number; TG210517-1, TG220518-1). Male C57/BL6 wild-type (AT1a^+/+^) mice were purchased from Japan SLC (Shizuoka, Japan). AT1a receptor knockdown homozygous (AT1a^−/−^) mice from a C57/BL6 background were generated as described previously^46^. Ten- to twelve-week-old male mice were used in the experiments. All mice had free access to laboratory chow (CRF-2; Charles River Laboratories Japan, Yokohama, Japan) and water. Body weights were measured before both denervation and tissue harvesting. Muscle strength was measured using the limb grip test with a grip strength meter (MK-380CM/FM; Muromachi Kikai Co., Ltd., Tokyo, Japan) before denervation. The average of three measurements of muscle strength per animal per time point was recorded for comparative analysis. All mice were housed in the Institute for Animal Experimentation at the St. Marianna University School of Medicine under controlled temperature (24 °C) and a 12-h light/dark cycle.

### Experimental design

Denervation in the Den group was performed by transection of each sciatic nerve exposed after skin incision in both right and left hind limbs under inhalation anesthesia with 2% isoflurane. The skin incision was closed using 4–0 nylon sutures. The mice were sacrificed immediately after euthanasia by intraperitoneal anesthesia with 400 mg/kg pentobarbital at 3 days (AT1a^+/+^, n = 9; AT1a^−/−^, n = 10), 7 days (AT1a^+/+^, n = 7; AT1a^−/−^, n = 6), and 21 days (AT1a^+/+^, n = 7; AT1a^−/−^, n = 6) postdenervation. Sham operation as a Cont group was performed in a similar manner without the nerve transection. The mice of a Cont group were sacrificed at 3 days (AT1a^+/+^, n = 9; AT1a^−/−^, n = 9), and 21 days (AT1a^+/+^, n = 6; AT1a^−/−^, n = 5). Serum samples and leg muscle tissues were collected from mice that were sacrificed at 3 days (AT1a^+/+^, n = 18; AT1a^−/−^, n = 19), 7 days (AT1a^+/+^, n = 7; AT1a^−/−^, n = 6), and 21 days (AT1a^+/+^, n = 13; AT1a^−/−^, n = 11). The extracted muscles specimens were sectioned into the gastrocnemius and TA muscle as a fast muscle and the soleus muscle as a slow muscle. After weighing, each muscle was frozen individually in liquid nitrogen and stored at − 80 °C for further analyses.

### Immunohistological analysis in muscle tissues

The frozen excised gastrocnemius, TA, and soleus muscle tissues were cut centrally along the width and embedded in Tissue-Tek® O.C.T.™ Compound (Sakura Finetek Japan Co., Ltd., Tokyo, Japan). Frozen sections sliced to a thickness of 10 μm were prepared using a cryostat (HM 550; Thermo Fisher Scientific, Waltham, MA, USA). Double-color immunofluorescent staining was performed to evaluate the diameter of each type IIb and I fiber as described previously^47^; briefly, the dried sections were incubated with the following primary antibodies at 4 °C overnight: anti-type IIb MyHC isoform immunoglobulin (Ig)G1 (MyHCIIb; 1 : 100; F18; Developmental Studies Hybridoma Bank (DSHB), the University of Iowa, Iowa City, IA, USA), anti-type I myosin heavy chain (MyHC) isoform IgG2b (MyHCI; 1 : 200; BA-F8; DSHB), and anti-laminin IgG (H + L) (1 : 3000; NB300-144; Novus Biologicals, Centennial, CO, USA). After washing three times with phosphate-buffered saline (PBS), the sections were incubated with the following fluorescent-labeled secondary antibodies at room temperature for 1 h: Alexa Flour 568-labeled goat anti-mouse IgG1 (1 : 500; Invitrogen Corporation, Carlsbad, CA, USA), Alexa Flour 488-labeled goat anti-mouse IgG2b (1 : 500; Invitrogen Corporation), and Alexa Flour 488-labeled goat anti-rabbit IgG (H + L) (1 : 500; Invitrogen Corporation). For quantification of the cross-sectional areas of the muscle fibers, cross-sectional areas of the specimens were evaluated using 300 fibers of type IIb muscle fibers or 100 fibers of type I muscle fibers at 100 × magnification per animal in a WinRoof image analyzer, version 4.3.0 (Mitani, Tokyo, Japan).

### Terminal deoxynucleotidyl transferase-mediated dUTP nick-end-labeling staining in muscle tissues

In the frozen sections of the gastrocnemius muscle at 21 days postdenervation sliced to a thickness of 10 μm, the TUNEL staining was performed using the Click-iT™ Plus TUNEL Assay for In Situ Apoptosis Detection, Alexa Fluor™ 594 dye (Thermo Fisher Scientific). Thereafter, to determine the location of the nuclei, the sections stained with the laminin were made by incubations of the anti-laminin IgG (H + L) antibody at 4 °C overnight and then the fluorescent-labeled secondary antibody (Alexa Flour 488-labeled goat anti-rabbit IgG (H + L) at room temperature for 1 h, as described above. Finally, the slides were cover-slipped with ProLong™ Diamond Antifade Mountant with DAPI (Thermo Fisher Scientific) for detection of all the nuclei in each section. For quantification of the TUNEL-positive nuclei, the cross-sectional areas of the specimens were evaluated in ≥ 6 visual fields at 200–400 × magnification per animal. The numbers of each TUNEL- and DAPI-positive nuclei were separately counted in the observed sections and a total of > 400 nuclei in ≥ 6 fields of view were counted for each section. The ratio of TUNEL-positive nuclear to all the nuclei (TUNEL- or DAPI-positive nuclei) was calculated.

### Assessment of macrophage content and phenotype in muscle tissues

To determine macrophage infiltration, we used the frozen sections of the gastrocnemius muscle at 21 days postdenervation sliced to a thickness of 10 μm. The dried sections were fixed with 4% paraformaldehyde in PBS for 15 min and immersed in 0.3% hydrogen peroxide solution for 20 min to inactivate the endogenous peroxidase activity. After a wash with PBS, the primary antibodies against F4/80 (rat monoclonal; 1:500, Bio-Rad, Hercules, CA, USA) and CD206 (rabbit polyclonal; 1:1000; Abcam, Cambridge, United Kingdom) were reacted at 4 °C overnight. Visualization was performed by incubation with polymeric horseradish peroxidase–conjugated secondary antibodies (ImmPRESS HRP Goat Anti-Rat IgG, Mouse adsorbed Polymer Detection Kit, Vector Laboratories, Newark, CA, USA) and fluorescent-labeled secondary antibodies (Alexa Flour 488-labeled goat anti-rabbit IgG (H + L); 1:1000, Invitrogen Corporation). Peroxidase activity was detected via the diaminobenzidine reaction (Liquid DAB+; DAKO Japan, Tokyo, Japan), and sections were counterstained with hematoxylin. For quantification of the macrophage infiltration, the number of cells was counted in six fields of view at 400 × magnification per animal.

### Real-time quantitative PCR

Frozen gastrocnemius muscle was homogenized using Sepasol-RNA I Super G (Nacalai Tesque, Inc., Kyoto, Japan), total RNA was extracted using an RNeasy Fibrous Mini kit (Qiagen, Valencia, CA, USA), and TaqMan real-time polymerase chain reaction with a StepOnePlus real-time polymerase chain reaction system (Thermo Fisher Scientific) was used to measure the mRNA levels of *MuRF1*(Mm01185221_m1)*, Atrogin1* (Mm00499523_m1)*, Bax* (Mm00432051_m1)*, Bcl-2* (Mm00477631_m1)*, C1qa* (Mm07295529_m1)*, Axin2* (Mm00443610_m1), *Agtr1a* (Mm01957722_s1), *angiotensinogen* (Mm0599662_m1) and *18S* rRNA (Mm03928990_g1).

### Western blot analysis

Protein samples extracted from frozen gastrocnemius muscle (15 µg) were separated by sodium dodecyl sulfate–polyacrylamide gel electrophoresis using NuPAGE 4–12% Bis–Tris gels and the XCell SureLock Mini-Cell system (Thermo Fisher Scientific) as described previously^48^. After blocking, the membranes were cut around molecular weight of each targeted molecule and were incubated overnight at 4 °C with primary antibodies against FoxO1 (rabbit monoclonal; #9454; 1:1000; Cell Signaling Technology, Danvers, MA, USA), phospho-FoxO1(Ser256) (rabbit monoclonal; #9461; 1:1000; Cell Signaling Technology), NF-κB p65 (rabbit monoclonal; #8242; 1:1000; Cell Signaling Technology), and phospho-NF-κB p65 (Ser536) (rabbit monoclonal; #3033; 1:1000; Cell Signaling Technology). We also used a rabbit monoclonal antibody to α-tubulin (ab176560; 1:4000, Abcam) to detect α-tubulin on the same membranes. After incubation with horseradish peroxidase-conjugated anti-rabbit antibody (ab97051; Abcam), protein bands were detected by chemiluminescence using the ECL Prime western blotting detection reagent (GE Healthcare, Chicago, IL, USA). Images were acquired on a charge-coupled device camera system (ImageQuant LAS 4000; GE Healthcare). The ratio of phosphorylated to total proteins was calculated using ImageJ software (NIH). The membranes used for immunoblotting against FoxO1 and phospho-FoxO1 were reused after treatment with stripping buffer (ATTO WSE-7240 EzReprobe, ATTO Corp., Tokyo, Japan) and incubated with the anti-α-tubulin antibody. The expression levels of all proteins were quantified using ImageJ software. Samples from the same experiment were processed in parallel for SDS polyacrylamide gel electrophoresis (SDS-PAGE) and western blotting using different gels and membranes, and the image data obtained were cropped. Entire images of western blotting are shown in online supplementary resource Figs. S5–S6.

### Measurement of serum complement C1q by ELISA

Serum complement C1q concentration was measured using a C1q, mouse, ELISA kit (Hycult Biotech, Uden, The Netherlands), which was the same as the kit used in the previous study showing the beneficial effects of Ang II receptor blockade against muscle injury^16^.

### Measurement of MCP-1 in gastrocnemius muscle by ELISA

Muscular MCP-1 in the proteins extracted from gastrocnemius muscle were measured by ELISA (R&D Systems, Minneapolice, MN, USA). Their values were normalized to total protein concentration.

### Statistical analysis

Data were expressed as the means ± standard errors, and statistical significance was accepted for *P* < 0.05. Following the Kruskal–Wallis test, differences within each group were identified using the Steel–Dwass test, and the differences between groups were compared using the unpaired Student’s *t*-test with JMP® software version 15.2.0 (SAS Institute, Cary, NC, USA).

## Supplementary Information

Supplementary Information.

## Data Availability

The data used to support the findings of this study are included within the article.
