# Supplementary material for: Angiotensin II type 1a receptor deficiency alleviates muscle atrophy after denervation
Source: Sci Rep. 2023 Jan 10;13:519. doi: 10.1038/s41598-023-27737-7 (PMC9832142; doi:10.1038/s41598-023-27737-7)
Supplement: Supplementary file 1 — Supplementary Information. [file 41598_2023_27737_MOESM1_ESM.pdf]

Angiotensin II type 1a receptor deficiency alleviates muscle atrophy after denervation

Short Title: Angiotensin II type 1a receptor and muscle atrophy

Suguru Takayama[1], Kazuho Inoue[2], Yuji Ogura[3], Seiko Hoshino[2], Takeshi Sugaya[1], Keiichi Ohata[1], Hitoshi Kotake[1], Daisuke Ichikawa[1], Minoru Watanabe[4], Kenjiro Kimura[5], Yugo Shibagaki[1], and Atsuko Kamijo-Ikemori[1,2,4,*]

[1]Division of Nephrology and Hypertension, Department of Internal Medicine, St. Marianna University School of Medicine, Kanagawa, Japan.

[2]Department of Anatomy, St. Marianna University School of Medicine, Kanagawa, Japan.

[3]Department of Physiology, St. Marianna University School of Medicine, Kanagawa, Japan.

[4]Institute for Animal Experimentation, St. Marianna University Graduate School of Medicine, Kanagawa, Japan.

[5]JCHO Tokyo Takanawa Hospital, Tokyo, Japan.

[*]Corresponding author

Correspondence should be addressed to: Atsuko Kamijo-Ikemori, M.D., Ph.D.,
Division of Nephrology and Hypertension, Department of Internal Medicine,
Department of Anatomy, and Experimental Animals Institution, St. Marianna University School of Medicine, 2-16-1 Sugao, Miyamae-Ku, Kawasaki 216-8511, Japan.
Tel: +81-44-977-8111 ext. 3630; Fax: +81-44-976-7083
E-mail: a2kamijo@marianna-u.ac.jp

Additional Figures

Supplementary Table S1 and S2.

AT1a receptor loss was associated with hyperphagia and obesity with increased adiposity

Method and Result

In order to measure food intake and organ weight, an additional experiment was performed in ten-week-old male AT1a$^{-/-}$ mice (n=13) and AT1a$^{+/+}$ mice (n=10). The mice were divided into two groups: the Den group (AT1a$^{-/-}$ mice: n=7, AT1a$^{+/+}$ mice: n=5) received the operation that their sciatic nerves in right and left inferior limbs were cut. The control mice (AT1a$^{-/-}$ mice: n=6, AT1a$^{+/+}$ mice: n=5) underwent sham operation.

Body weight levels were significantly higher in all AT1a$^{-/-}$ mice than in all AT1a$^{+/+}$ mice (Table S1), the mean values of food intake were higher in AT1a$^{-/-}$ -Cont group than in AT1a$^{+/+}$ -Cont group, but not significantly, and weights of adipose tissue in addition to kidney were significantly greater in all AT1a$^{-/-}$ mice than in all AT1a$^{+/+}$ mice, but not liver, heart, and lung (Table S2). In addition, the mean values of food intake after denervation were lower in AT1a$^{-/-}$ -Den group than in AT1a$^{+/+}$ -Den group (Table S1) and the greater food intake after denervation was not observed in the AT1a$^{-/-}$ -Den group compared to the AT1$^{+/+}$ -Den group. On the other hand, the weight of adipose tissue after denervation in the AT1a$^{-/-}$ -Den group was significantly decreased than the AT1a$^{-/-}$ -Cont group.

Table S1. Body weight and food intake during an observational period.

	AT1a$^{-/-}$ -Cont (n=6)	AT1a$^{-/-}$ -Den (n=7)	AT1a$^{+/+}$ -Cont (n=5)	AT1a$^{+/+}$ -Den (n=5)
Body weight (g)				
Pre-operation	27.3 ± 0.6$^{\#\#}$	26.7 ± 0.7§§	23.6 ± 0.6	24.5 ± 0.3
7days	27.8 ± 0.5$^{\#\#}$	26.5 ± 0.6§§	24.1 ± 0.6	25.3 ± 0.2
14days	28.2 ± 0.5$^{\#\#}$	27.0 ± 0.6§§	24.8 ± 0.5	25.4 ± 0.3
21days	28.6 ± 0.5$^{\#\#}$	27.4 ± 0.6§§	25.2 ± 0.4	25.8 ± 0.3

Food intake (g)				
Pre-operation	3.9 ± 0.1	3.5 ± 0.1	3.2 ± 0.2	3.7 ± 0.1
7days	3.7 ± 0.1	4.0 ± 0.2	3.5 ± 0.2	4.1 ± 0.2
14days	3.8 ± 0.1	3.8 ± 0.1	3.6 ± 0.2	4.3 ± 0.3
21days	4.0 ± 0.2	3.9 ± 0.1	3.5 ± 0.2	4.4 ± 0.3

Values are mean ± SE. $^{\#\#}$ *P* <0.01 vs AT1a$^{+/+}$ -Cont; §§*P* <0.01 vs AT1a$^{+/+}$ -Den.

Table S2. Weigh of organs at 21 days post denervation.

	AT1a$^{-/-}$ -Cont (n=6)	AT1a$^{-/-}$ -Den (n=7)	AT1a$^{+/+}$ -Cont (n=5)	AT1a$^{+/+}$ -Den (n=5)
Liver	1.03 ± 0.05	0.99 ± 0.05	0.99 ± 0.06	1.03 ± 0.03
Adipose tissue	0.57 ± 0.02$^{\#\#}$	0.46 ± 0.03*, §§	0.27 ± 0.03	0.25 ± 0.02
kidney	0.39 ± 0.01$^{\#\#}$	0.36 ± 0.01§§	0.30 ± 0.01	0.30 ± 0.01
Heart	0.13 ± 0.00	0.14 ± 0.00	0.12 ± 0.00	0.14 ± 0.01
Lung	0.16 ± 0.01	0.16 ± 0.00	0.15 ± 0.00	0.16 ± 0.01

Values are mean ± SE. **P*<0.05 vs the same group control; $^{\#\#}$ *P* <0.01 vs AT1a$^{+/+}$ -Cont; §§*P* <0.01 vs AT1a$^{+/+}$ -Den.

Supplementary Figure S1.

Pharmacological blockade of AT1 receptor, losartan, alleviated denervation-induced muscle atrophy

Method and Result

The beneficial effects of AT1 receptor blocker, losartan, against the denervated muscle atrophy was evaluated using ten-week-old male AT1a$^{+/+}$ mice. The AT1a$^{+/+}$ mice (n=16) were divided into three groups: the Den group (n=5, 23-24.5 g) received the operation that their sciatic nerves in right and left inferior limbs were cut. The DEN + losartan group (n=6, 22.6-23.7 g) was subjected to losartan (Sigma-Aldrich, St. Louis, MO, USA) ad libitum in their water (0.5 g/L, Sigma) in drinking water in addition to the denervation, while the control mice (n=5, 22.6-24.8 g) underwent sham operation. Treatment of losartan was started one week before the procedure of the denervation to 21 days postdenervation. The dose of losartan used in this study was reported to exert the protective effect against the muscle injury by gastrocnemius lacerations [1]. As a

results, the cross-sectional areas of type IIb muscle fibers in gastrocnemius muscle decreased at 21 days postdenervation in both Den group and Den + losartan group, and the reduction was significantly attenuated in the denervated muscles of Den + losartan group compared to the Den group. These results supported the results obtained in AT1a$^{-/-}$ mice.

Figure S1

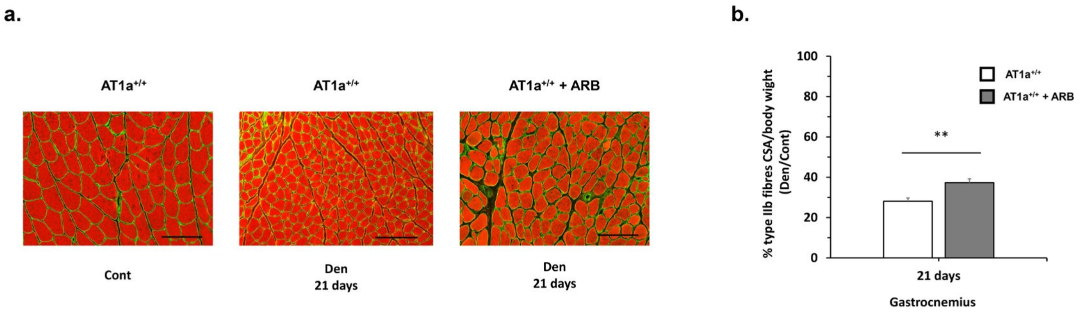

Figure S1 legend

Immunohistochemistry analysis of muscle fibers (a, b). Triple staining with type IIb muscle fibers stained red, type I muscle fibers stained green, and laminin stained green (a). Cross-section of muscle fibers in gastrocnemius (b). Scale bar: 100 μm. The graphs show the fold decrease in each cross-sectional area normalized to body weight in denervated muscle (Den group) compared to sham-operated group as control (Cont group). Cont group, n = 5; Den group, n = 5; Den + losartan group, n = 6. Values are means ± SE. $^{**}P < 0.01$.

Supplementary Figure S2.

AT1a receptor loss did not activate muscular protein synthesis in denervated gastrocnemius muscles

Results

To evaluate activation of muscular protein synthesis, activations of Akt (Fig. S2a-d), mTOR (Fig. S2e-h), and S6K (Fig. S2i-l) were evaluated by western blot analysis in the gastrocnemius muscle of the mice described in the manuscript. Protein expression levels in each sample were normalized to α-tubulin expression levels and shown as the fold

increase or decrease in protein expression in the Den groups compared with the Cont groups. The ratios of phosphorylated Akt, mTOR, and S6K to total each protein were not significantly different between the AT1a$^{+/+}$ and AT1a$^{-/-}$ mice.

Method

Western blot analysis

Protein samples extracted from frozen gastrocnemius muscle (15 μg) were separated by sodium dodecyl sulfate-polyacrylamide gel electrophoresis using NuPAGE 4%–12% Bis-Tris gels and the XCell SureLock Mini-Cell system (Thermo Fisher Scientific, Waltham, MA, USA) as described previously. After blocking, the membranes were were cut around molecular weight of each targeted molecule and were incubated overnight at 4°C with primary antibodies against Akt (rabbit monoclonal; #9272; 1:1000; Cell Signaling Technology, Danvers, MA, USA), phospho-Akt (Ser 473) (rabbit monoclonal; #9271; 1:1000; Cell Signaling Technology), mTOR(rabbit monoclonal; #2983; 1:1000; Cell Signaling Technology), phospho-mTOR (Ser2448) (rabbit monoclonal; #5536; 1:1000; Cell Signaling Technology), S6K(rabbit monoclonal; #2708; 1:1000; Cell Signaling Technology), phospho-p70S6 kinase (S6K; rabbit monoclonal; #9234; 1:1000; Cell Signaling Technology). We also used a rabbit monoclonal antibody to α-tubulin (ab176560; 1:4000, Abcam, Cambridge, United Kingdom) to detect α-tubulin on the same membranes. After incubation with horseradish peroxidase-conjugated anti-rabbit antibody (ab97051; Abcam), protein bands were detected by chemiluminescence using the ECL Prime western blotting detection reagent (GE Healthcare, Chicago, IL, USA). Images were acquired on a charge-coupled device camera system (ImageQuant LAS 4000; GE Healthcare). The ratio of phosphorylated to total proteins was calculated using ImageJ software (National Institutes of Health (NIH), Bethesda, MD, USA). The expression levels of all proteins were quantified using ImageJ software (NIH). Entire images of western blot analysis are shown in a Supplementary Information file (Supplementary Fig. S7–9).

Figure S2

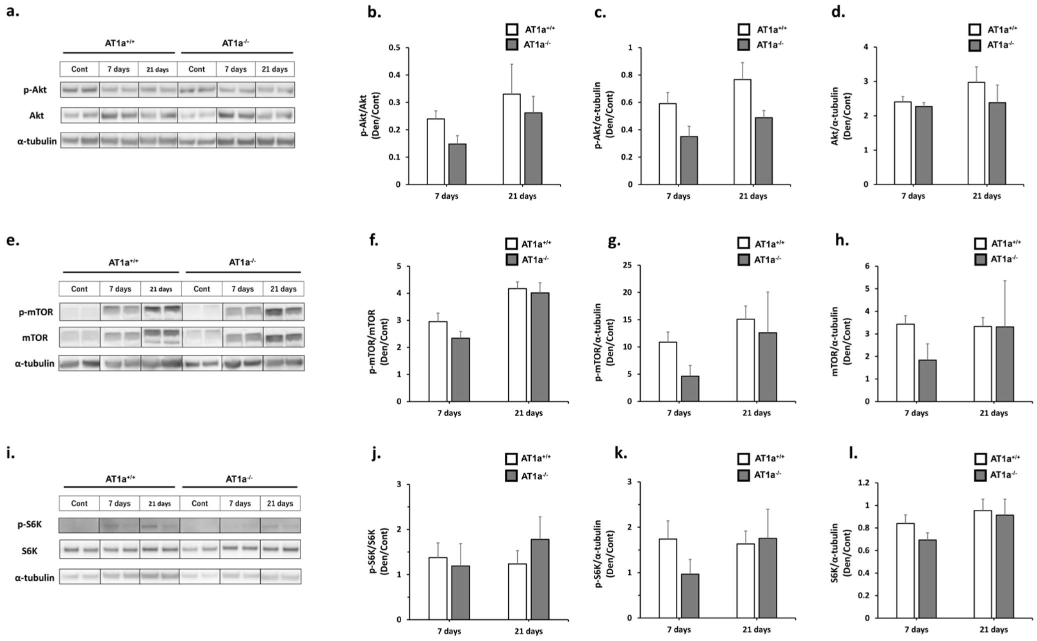

Figure S2 legend

Protein expression levels of phosphorylated Akt, mTOR, and S6K and total Akt, mTOR, and S6K were evaluated by western blot analysis (p-Akt/Akt/α-tubulin, a-d; p-mTOR/mTOR/α-tubulin, e-h; p-S6K/S6K/α-tubulin, i-l). Expression levels in each sample were normalized to α-tubulin expression levels and were shown as the fold increase or decrease in the protein expression in the Den group compared with the Cont group. Relative protein expression was calculated for p-Akt/ Akt (b), p-Akt /α-tubulin (c), Akt /α-tubulin (d), p-mTOR/ mTOR (f), p- mTOR /α-tubulin (g), mTOR /α-tubulin (h), p-S6K/ S6K (j), p- S6K /α-tubulin (k), S6K /α-tubulin (l). Samples from the same experiment were processed in parallel for SDS-PAGE and western blotting using different gels and membranes, and the image data obtained were cropped. Entire images of western blotting are shown in online supplementary resource Supplementary Fig. S7-9. $AT1a^{+/+}$-Cont group, n = 6; $AT1a^{+/+}$-Den group at 7 days postdenervation, n = 7; $AT1a^{+/+}$-Den group at 21 days postdenervation, n = 7; $AT1a^{-/-}$-Cont group, n = 5; $AT1a^{-/-}$-Den group at 7 days postdenervation, n = 6; $AT1a^{-/-}$-Den group at 21 days postdenervation, n = 6. Values are means ± SE.

Supplementary Figure S3.

Mice without AT1a receptor did not upregulate the gene expressions of *Pax7, MyoD,* and *myogenin* more than mice with AT1a receptor in denervated gastrocnemius muscles

Method and Result

As AT1a receptor is expressed in satellite cells, the deficiency of AT1a receptor may influence on muscle repair function regulated by activation of muscle satellite cells. Therefore, the gene expressions of *Pax7* which is expressed in activate and proliferative muscle satellite cells, and both *MyoD* and *myogenin* which playcritical roles for the differentiation of myoblasts to myofibers in muscle tissue, were evaluated by RT-qPCR (*Pax7*, Mm01354484_m1; *MyoD1,* Mm01203489_g1; *myogenin,* Mm00446194_m1, Thermo Fisher Scientific) using the gastrocnemius muscle of the mice described in the manuscript. The expression levels of these transcripts in each sample were normalized to *18S* ribosomal RNA (*18S* rRNA, Mm03928990_g1, Thermo Fisher Scientific) expression levels and were shown as the fold increase or decrease in mRNA expression in the Den group compared with the Cont group. In the denervated gastrocnemius muscle, the gene expressions of *Pax7* at 21 days postdenervation (Fig. S3a), *MyoD* at 7 days and 21 days postdenervation (Fig. S3b), and *myogenin* at 7 days and 21 days postdenervation in AT1a$^{+/+}$ mice (Fig. S3c) were significantly upregulated compared with the Cont group and the gene expression of *myogenin* at 21 days postdenervation (Fig. S3c) in AT1a$^{-/-}$ mice was significantly upregulated compared with the Cont group. In the Den group, the gene expression of *Pax7* at 21 days postdenervation tended to be higher in the AT1a$^{+/+}$ mice than in the AT1a$^{-/-}$ mice (Fig. S3a) and the gene expressions of both *MyoD* and *myogenin* at 7 days and 21 days postdenervation were significantly upregulated in the AT1a$^{+/+}$ mice than in the AT1a$^{-/-}$ mice (Fig. S3b and S3c).

Figure S3

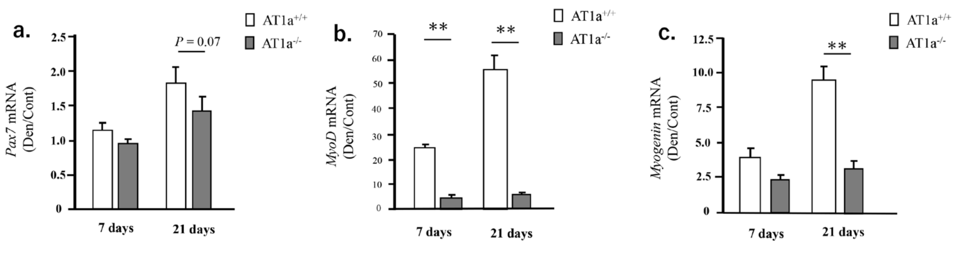

Figure S3 legend

Gene expressions of *Pax7* (a), *MyoD* (b), and *myogenin* (c) in gastrocnemius muscle were evaluated by RT-qPCR. Expression levels of these transcripts in each sample were normalized to *18S* rRNA expression levels and were shown as the fold increase or decrease in mRNA expression in the Den group compared with the Cont group. $AT1a^{+/+}$-Cont group, n = 6; $AT1a^{+/+}$-Den group at 7 days postdenervation, n = 7; $AT1a^{+/+}$-Den group at 21 days postdenervation, n = 7; $AT1a^{-/-}$-Cont group, n = 5; $AT1a^{-/-}$-Den group at 7 days postdenervation, n = 6; $AT1a^{-/-}$-Den group at 21 days postdenervation, n = 6. Values are means ± SE. ***P* < 0.01.

Supplementary Figure S4.

The degree of AT1a receptor gene expression in gastrocnemius muscle was significantly lower than those in kidney, heart, and liver

Method and Result

In order to reveal the degree of *AT1a receptor (Agtr1a*) expression in some organs including skeletal muscles, an additional experiment was performed using normal ten-week-old male $AT1a^{+/+}$ mice (n=6). Gastrocnemius muscle, tibialis anterior muscle, soleus muscle, kidney, heart, lung, liver, and brain were removed from the mice and were homogenized using Sepasol-RNA I Super G (Nacalai Tesque, Inc., Kyoto, Japan). Total RNA was extracted using an RNeasy Fibrous Mini kit (Qiagen, Venlo, Netherlands) and TaqMan real-time polymerase chain reaction with a StepOnePlus real-time polymerase chain reaction system (Thermo Fisher Scientific) was used to measure the mRNA levels of *Agtr1a* (Mm01957722_s1) and *18S* rRNA (Mm03928990_g1). The degree of *AT1a receptor* expression in gastrocnemius muscle was significantly lower than those in kidney, heart, and liver (Fig. S4).

Figure S4

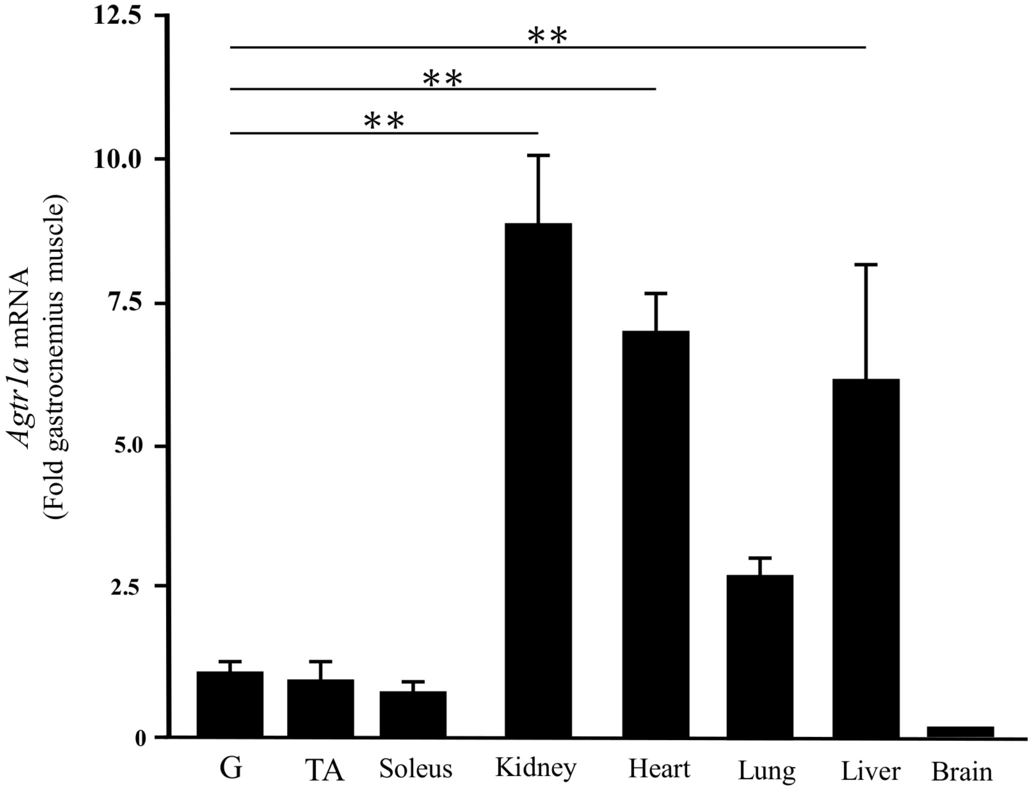

G; Gastrocnemius muscle, TA; Tibialis anterior muscle

Figure S4 legend

Gene expressions of *AT1a receptor* (*Agtr1a*) in gastrocnemius (G), tibialis anterior (TA), and soleus muscle, kidney, heart, lung, liver, and brain were evaluated by RT-qPCR in $AT1a^{+/+}$ mice (n=6). Expression levels of these transcripts in each sample were normalized to *18S* rRNA expression levels and were shown as the fold increase or decrease in mRNA expression in other organ compared with the gastrocnemius (G) muscle. Values are means ± SE. $^{**}P < 0.01$ vs gastrocnemius (G) muscle.

Entire images of western blot analysis

Supplementary Figure S5.

a. Phospho-FoxO1

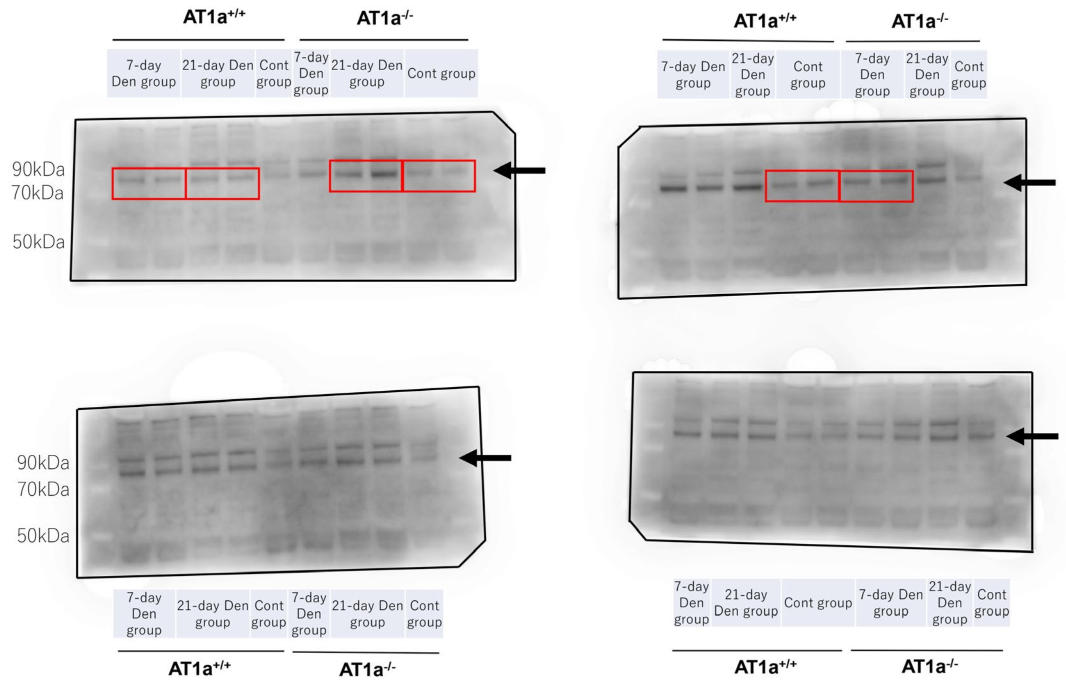

b. FoxO1

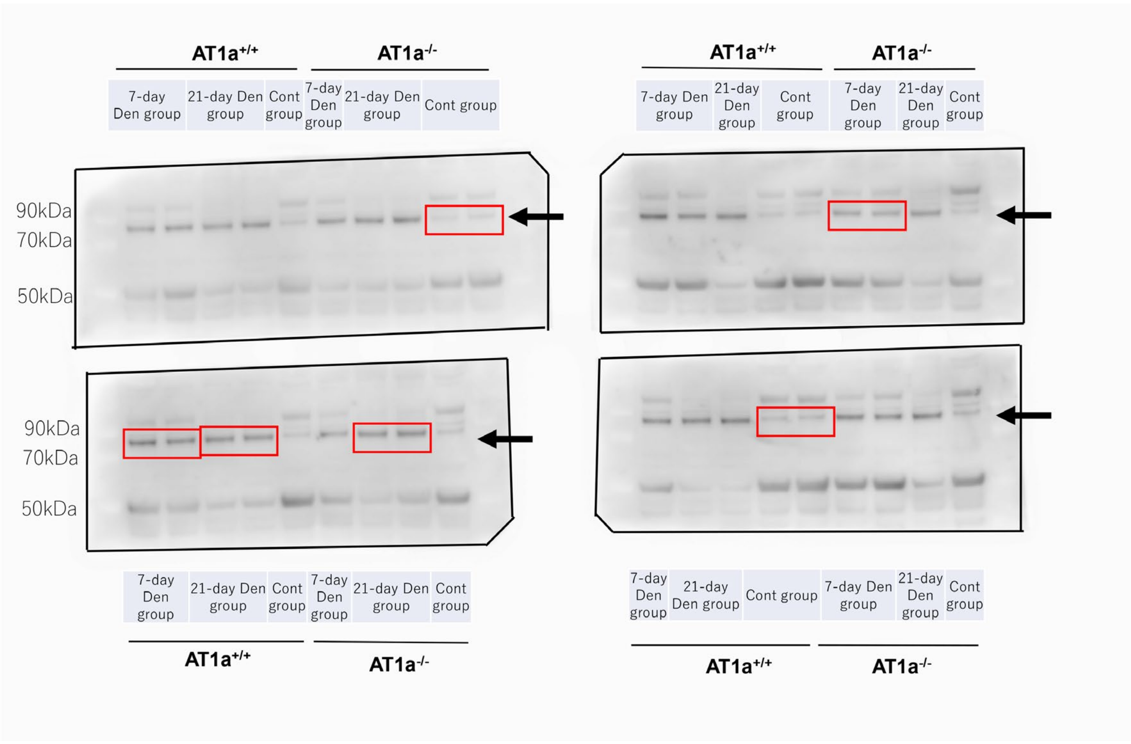

c. α-tubulin

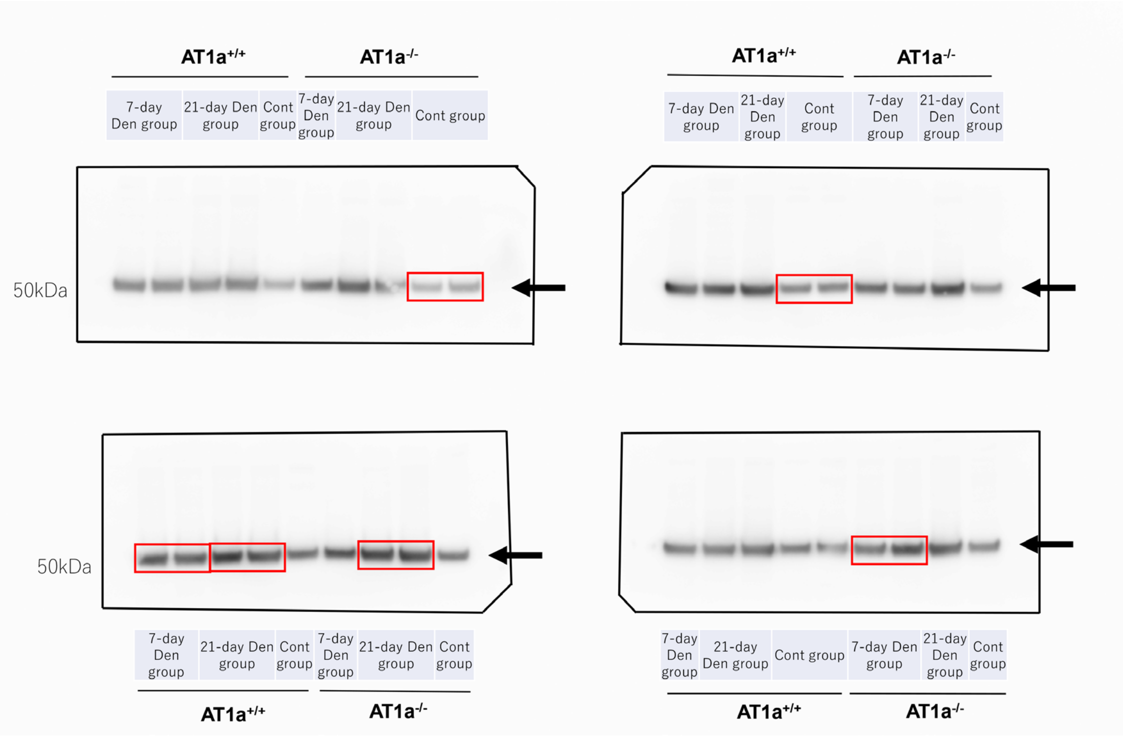

Western blot analysis of phospho-FoxO1 (a), FoxO1 (b), and α-tubulin (c) in the Den and Cont groups at 7 and 21days postdenervation. Black arrows represent the targeted protein. The black lines represent the edge of each cut membrane. Red boxes show the regions of the original blots used in main figures.

Supplementary Figure S6.

a. Phospho-NF-κB

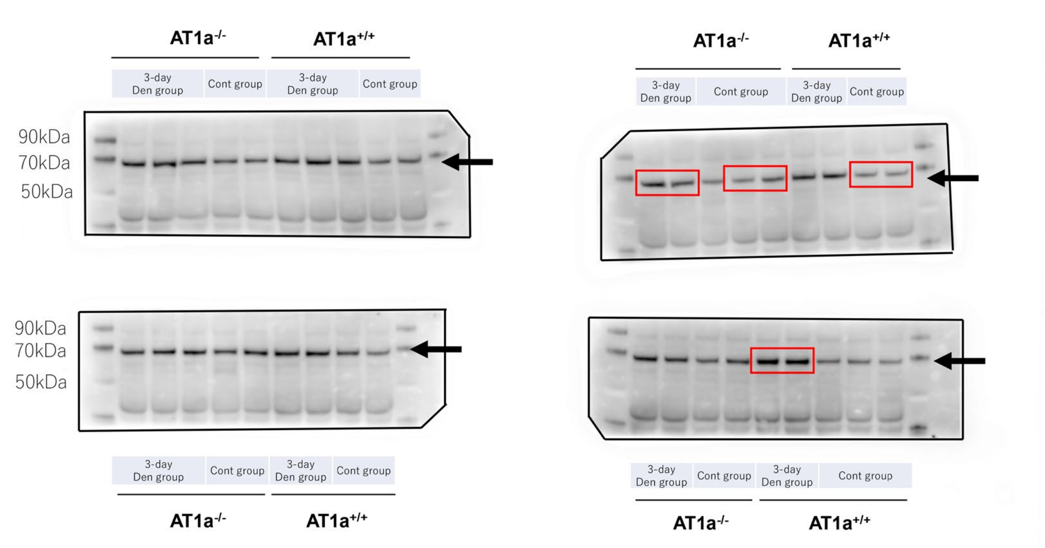

b. NF-κB

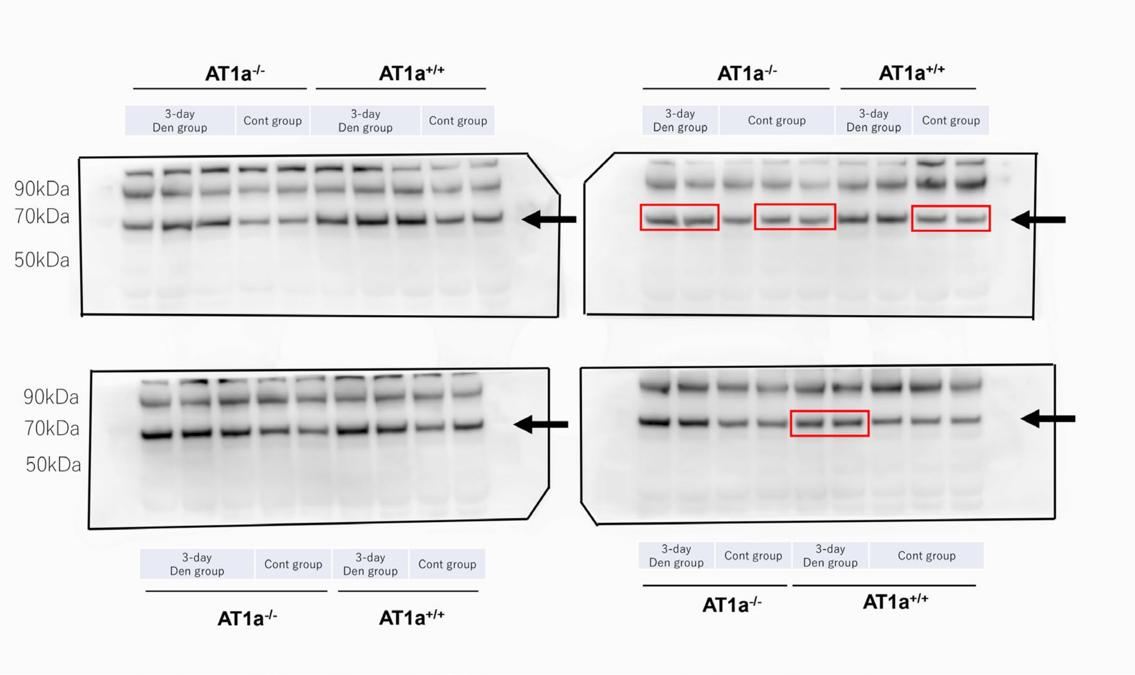

c. α-tubulin

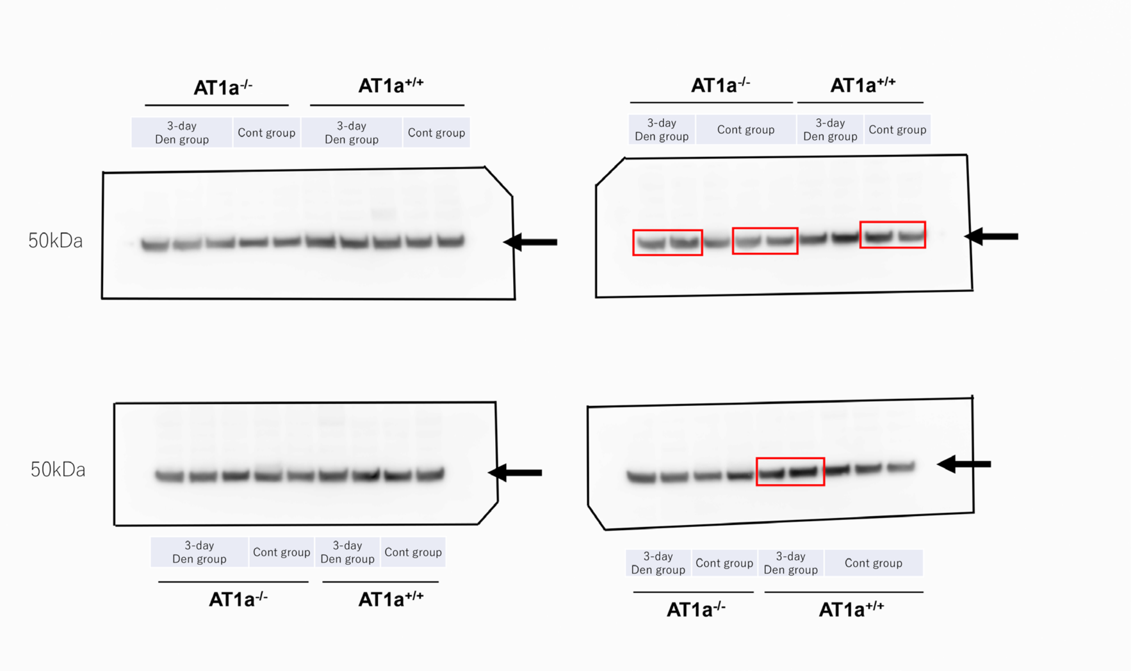

Western blot analysis of phospho-NF-κB (a), NF-κB (b), and α-tubulin (c) in the Den and Cont groups at 3 days postdenervation. Black arrows represent the targeted protein. The black lines represent the edge of each cut membrane. Red boxes show the regions of the original blots used in main figures.

Supplementary Figure S7.

a. Phospho-Akt

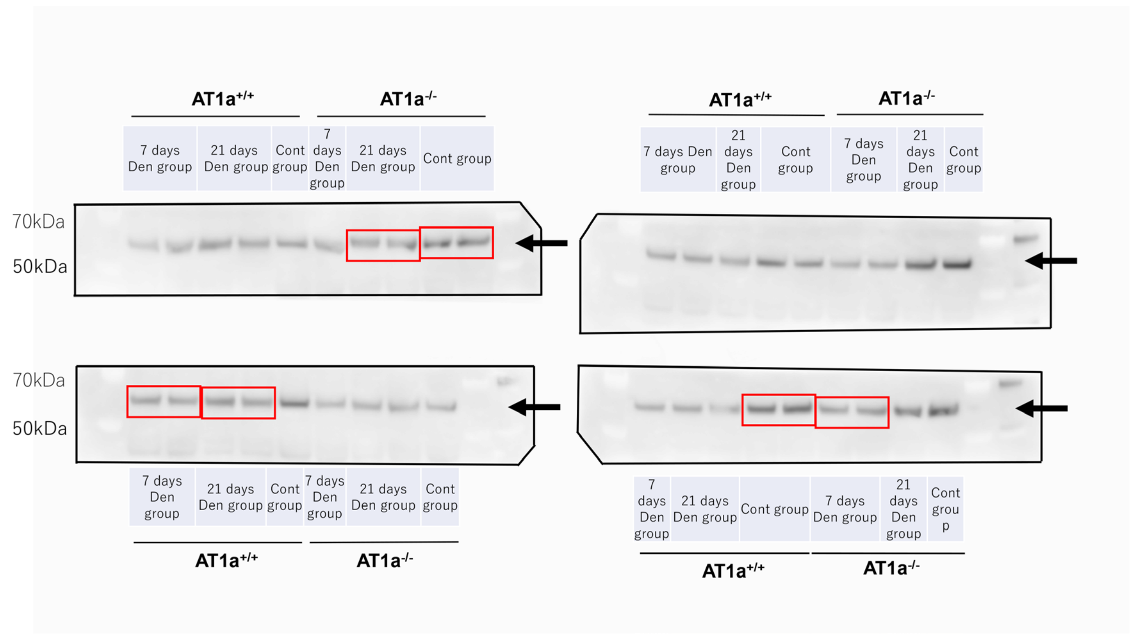

b. Akt

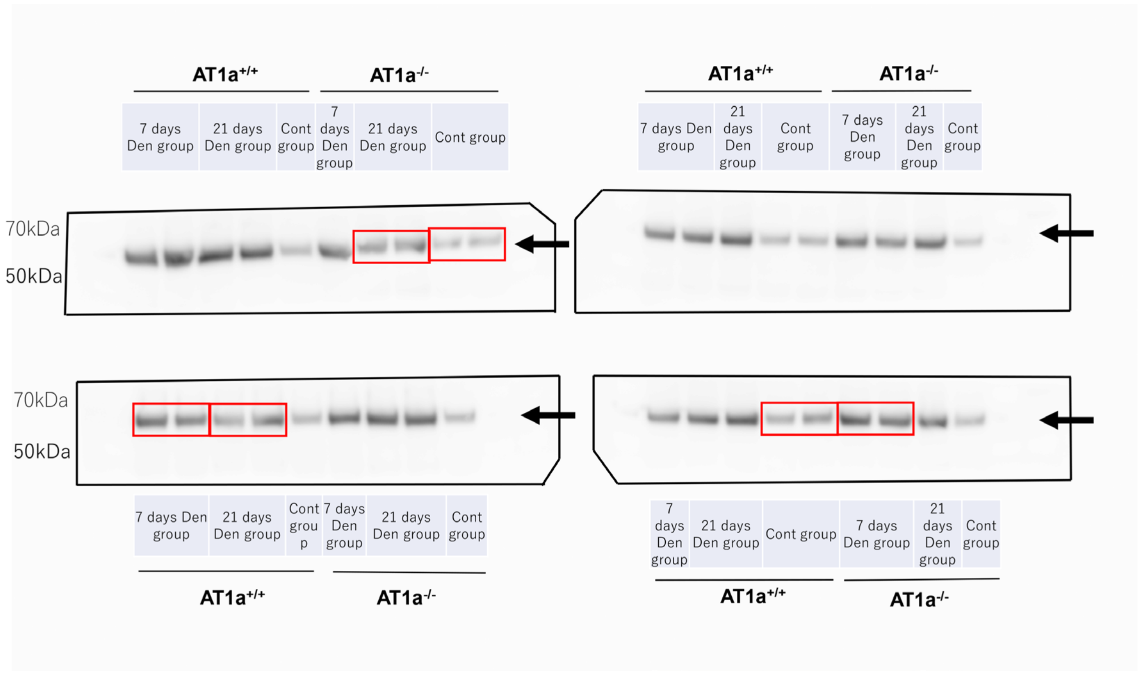

c. α-tubulin

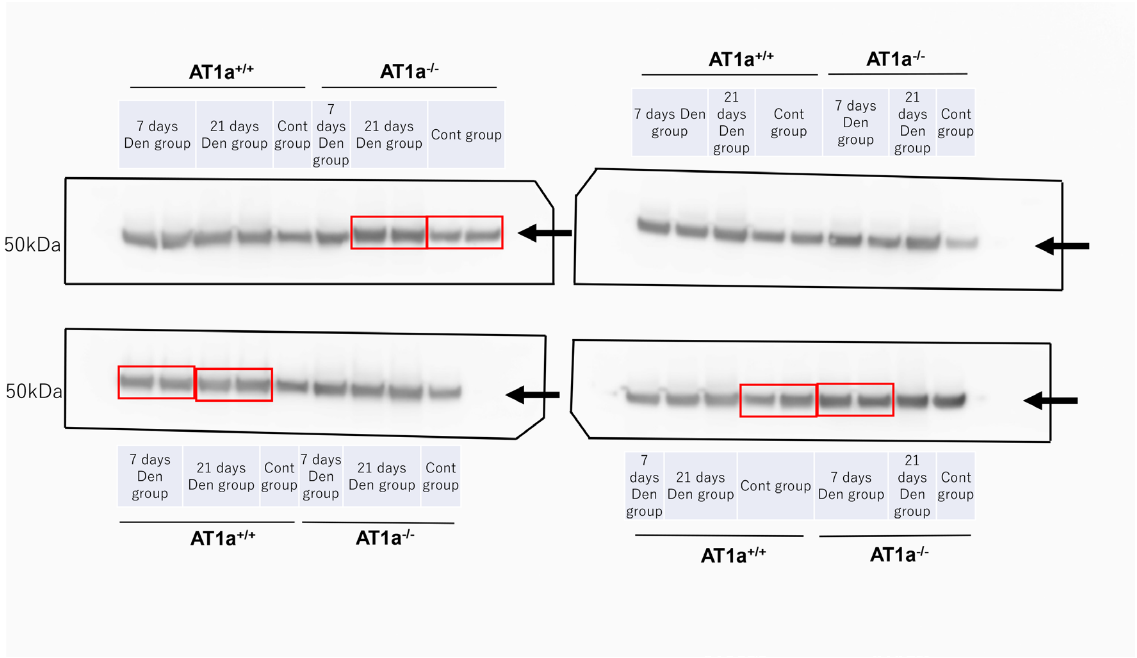

Western blot analysis of phospho-Akt (a), Akt (b), and α-tubulin (c) in the Den and Cont groups at 7 and 21days postdenervation. Black arrows represent the targeted protein. The black lines represent the edge of each cut membrane. Red boxes show the regions of the original blots used in main figures.

Supplementary Figure S8.

a. Phospho-mTOR

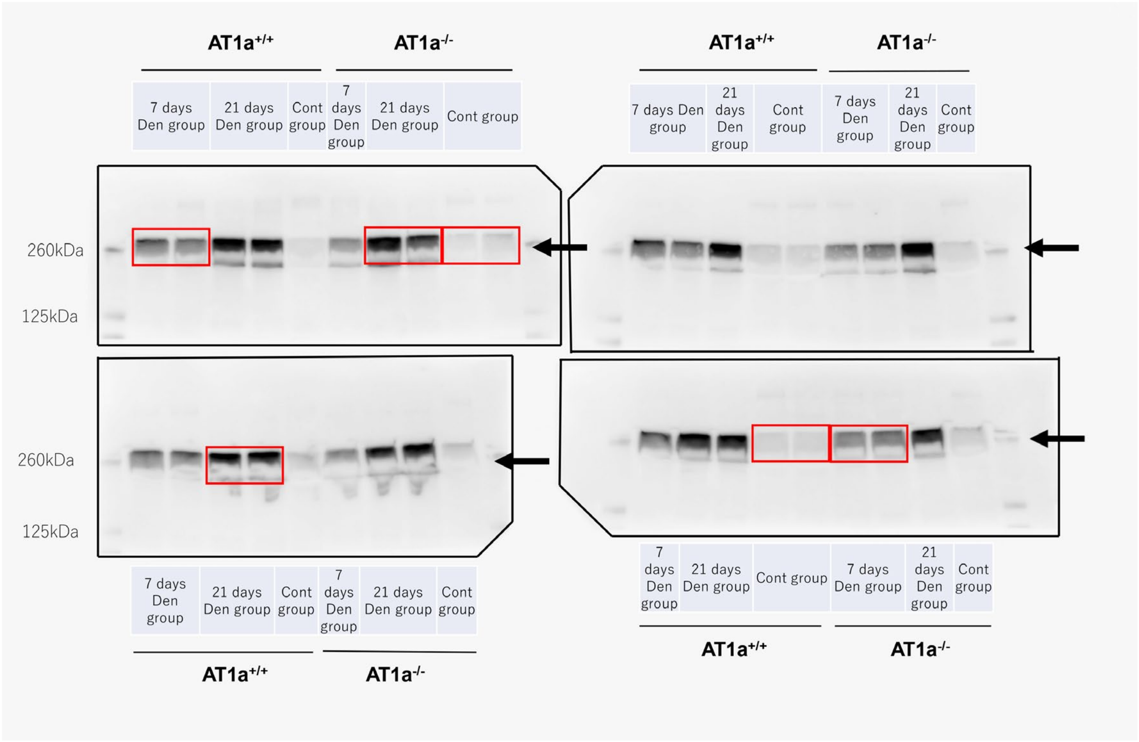

b. mTOR

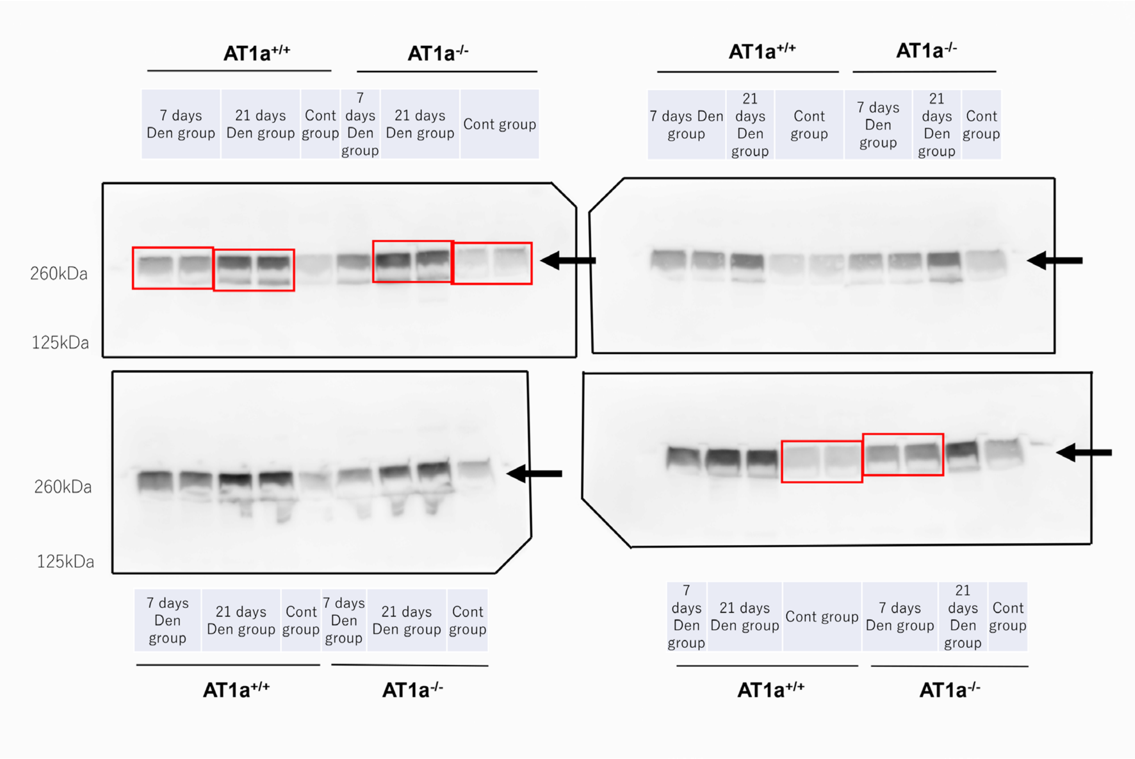

c. α-tubulin

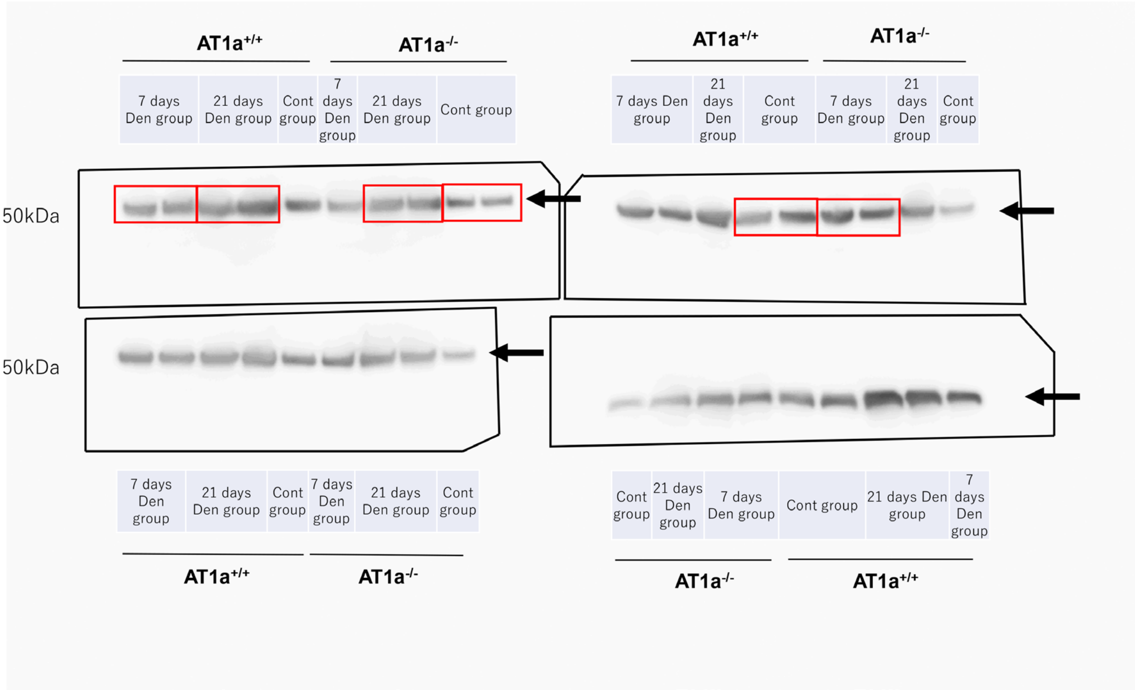

Western blot analysis of phospho-mTOR (a), mTOR (b), and α-tubulin (c) in the Den and Cont groups at 7 and 21 days postdenervation. Black arrows represent the targeted protein. The black lines represent the edge of each cut membrane. Red boxes show the regions of the original blots used in main figures.

Supplementary Figure S9.

a. Phospho-S6K

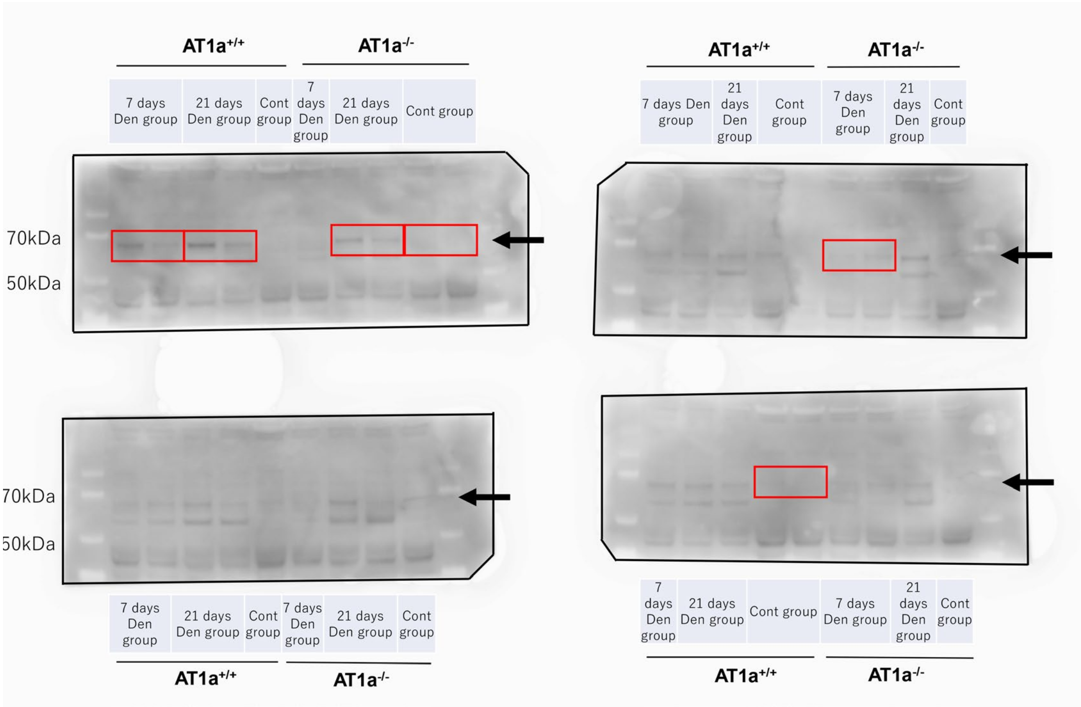

b. S6K

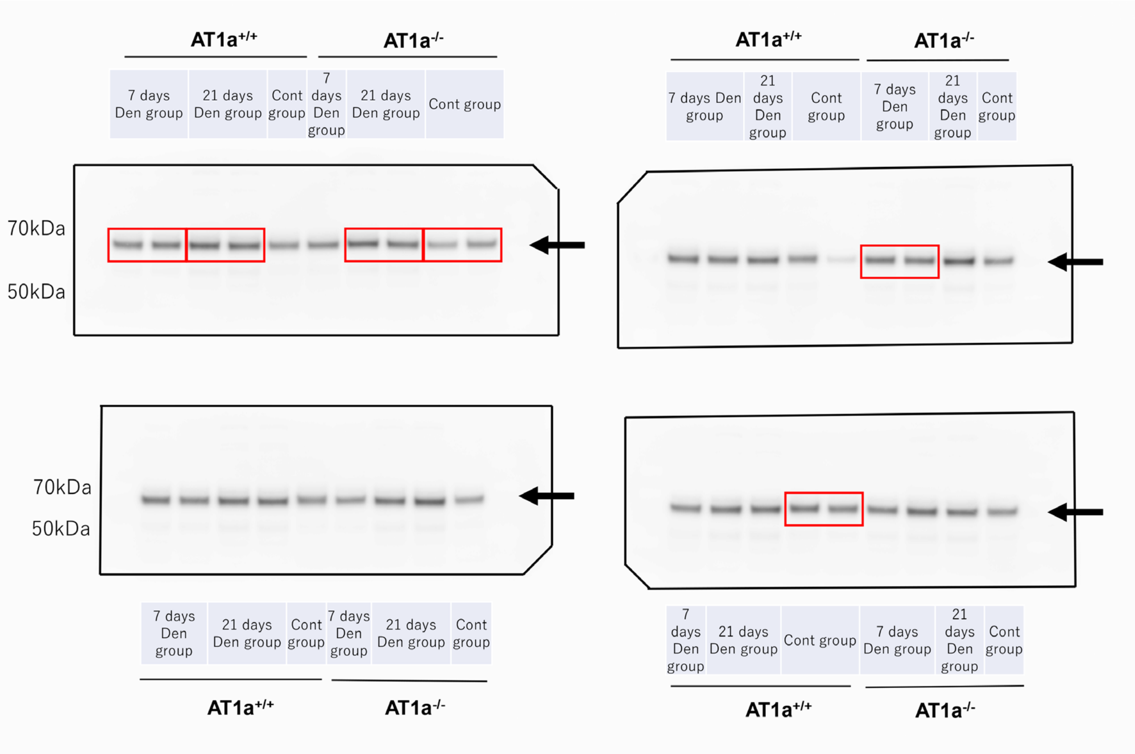

c. α-tubulin

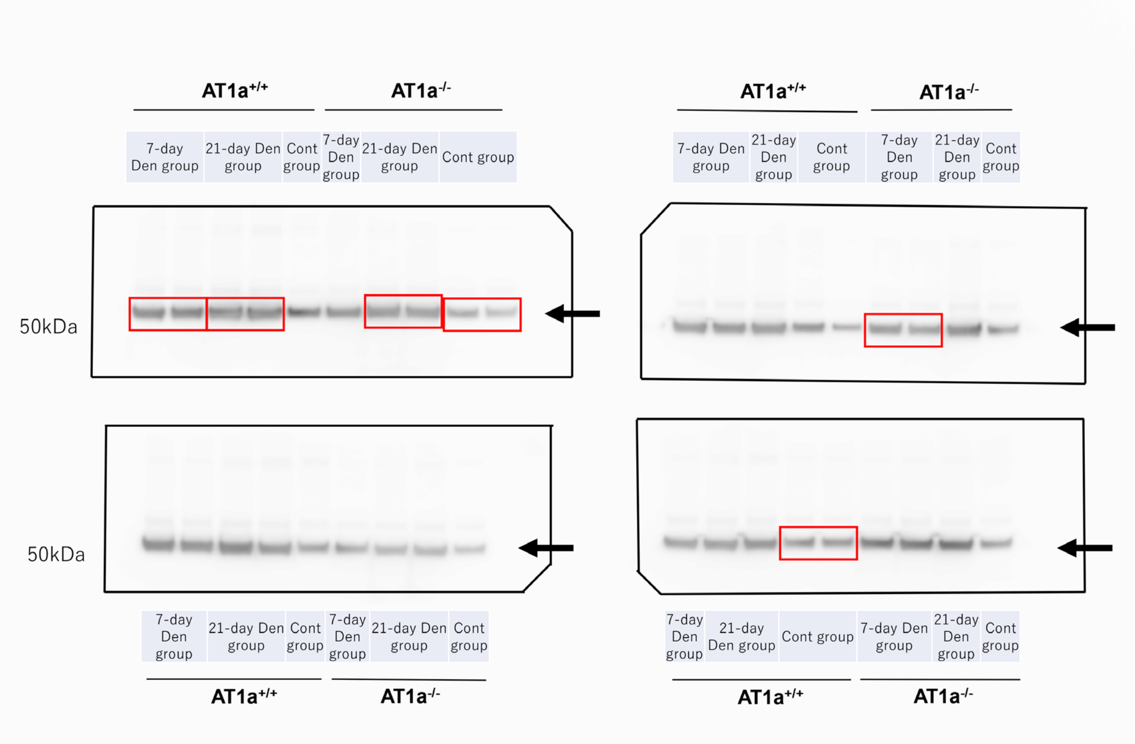

Western blot analysis of phospho-S6K (a), S6K (b), and α-tubulin (c) in the Den and Cont groups at 7 and 21 days postdenervation. Black arrows represent the targeted protein. The black lines represent the edge of each cut membrane. Red boxes show the regions of the original blots used in main figures.

References

1 Bedair, H. S., Karthikeyan, T., Quintero, A., Li, Y. & Huard, J. Angiotensin II receptor blockade administered after injury improves muscle regeneration and decreases fibrosis in normal skeletal muscle. *Am J Sports Med* **36**, 1548-1554, doi:10.1177/0363546508315470 (2008).
